# Reduced transmission of *Mycobacterium africanum* compared to *Mycobacterium tuberculosis* in urban West Africa

**DOI:** 10.1016/j.ijid.2018.05.014

**Published:** 2018-08

**Authors:** Prince Asare, Adwoa Asante-Poku, Diana Ahu Prah, Sonia Borrell, Stephen Osei-Wusu, Isaac Darko Otchere, Audrey Forson, Gloria Adjapong, Kwadwo Ansah Koram, Sebastien Gagneux, Dorothy Yeboah-Manu

**Affiliations:** a Noguchi Memorial Institute for Medical Research (NMIMR), University of Ghana, Legon, Ghana; b West African Centre for Cell Biology of Infectious Pathogens, University of Ghana, Legon, Ghana; c Department of Biochemistry, Cell and Molecular Biology, University of Ghana, Legon, Ghana; d Swiss Tropical and Public Health Institute, Basel, Switzerland; e University of Basel, Basel, Switzerland; f Korle-Bu Teaching Hospital, Korle-Bu, Accra, Ghana; g Centre for Plant Medicine Research, Mampong, Ghana

**Keywords:** *Mycobacterium tuberculosis*, *Mycobacterium africanum*, Transmission, Molecular epidemiology, MIRU-VNTR, Spoligotyping

## Abstract

**Objective:**

Understanding transmission dynamics is useful for tuberculosis (TB) control. A population-based molecular epidemiological study was conducted to determine TB transmission in Ghana.

**Methods:**

*Mycobacterium tuberculosis* complex (MTBC) isolates obtained from prospectively sampled pulmonary TB patients between July 2012 and December 2015 were characterized using spoligotyping and standard 15-locus mycobacterial interspersed repetitive unit variable number tandem repeat (MIRU-VNTR) typing for transmission studies.

**Results:**

Out of 2309 MTBC isolates, 1082 (46.9%) unique cases were identified, with 1227 (53.1%) isolates belonging to one of 276 clusters. The recent TB transmission rate was estimated to be 41.2%. Whereas TB strains of lineage 4 belonging to *M. tuberculosis* showed a high recent transmission rate (44.9%), reduced recent transmission rates were found for lineages of *Mycobacterium africanum* (lineage 5, 31.8%; lineage 6, 24.7%).

**Conclusions:**

The study findings indicate high recent TB transmission, suggesting the occurrence of unsuspected outbreaks in Ghana. The observed reduced transmission rate of *M. africanum* suggests other factor(s) (host/environmental) may be responsible for its continuous presence in West Africa.

## Highlights

• The estimated recent tuberculosis (TB) transmission rate (clustering rate of 41.2%) was found to be high in Ghana.
• There is a need for increased TB awareness by the national tuberculosis control program.
• *Mycobacterium africanum* (MAF) transmits at a lower rate compared to *Mycobacterium tuberculosis* in Ghana.
• The incidence of MAF remained fairly constant over the study years.
• Other factors may likely be responsible for maintaining MAF in West Africa.

## Introduction

Tuberculosis (TB) is a global health emergency; in 2016 an estimated 10.4 million people got sick, while 1.7 million died of TB (WHO, 2017). In 1993, the World Health Organization (WHO) declared TB a global health emergency and called for more efforts and resources to fight TB. Due largely to the inefficacy of the bacillus Calmette–Guérin (BCG) vaccine against pulmonary TB in adults, the current TB control strategy relies on case detection and treatment under the directly observed therapy short course (DOTs) strategy. The conventional indicators used to assess national control programs under this strategy focus on the proportion of cases that are cured at the end of treatment or whose sputum microscopy becomes negative after the first 2 months of treatment. Such indicators ignore equally important aspects of TB control, which include the duration of infectivity, the frequency of reactivation, and the risk of progression among the infected contacts, as well as the proportion of TB due to recent transmission.

Understanding transmission dynamics will contribute to knowledge on factors that enhance the spread of the disease, which is useful for developing preventive interventions. Molecular epidemiological studies have been very useful in a number of countries, identifying populations at risk and areas of high transmission, as well as providing much understanding on the prevalence of different *Mycobacterium tuberculosis* complex (MTBC) strains with varied virulence and drug resistance rates (Anderson et al., 2014, Malm et al., 2017, Seto et al., 2017, Varghese et al., 2013, Walker et al., 2014, Yang et al., 2016). These studies have shown that the dynamics of TB transmission vary greatly geographically. Even though Africa harbors a large proportion of the global TB cases, with a current incidence of 254 per 100 000 population (WHO, 2017), population-based molecular epidemiological studies needed to understand transmission patterns are rare. The few studies conducted have not been population-based and have lacked an in-depth analysis of the transmission dynamics of MTBC strains belonging to different lineages (Asante-Poku et al., 2016, Glynn et al., 2010, Mulenga et al., 2010).

The molecular typing tools – spacer oligonucleotide typing (spoligotyping) and mycobacterial interspersed repetitive unit variable number tandem repeat (MIRU-VNTR) typing – have been used successfully for strain differentiation in TB transmission studies due to their combined high discriminatory power and reproducibility; furthermore, in combination with epidemiological data, they have been used for the detection of recent TB transmission and outbreaks (Anderson et al., 2014, Barnes and Cave, 2003, Maguire et al., 2002, Surie et al., 2017, Varghese et al., 2013). Currently, the high cost and expertise needed for whole genome sequencing and analysis have precluded its use in population-based studies, and considering capacity building in a low-resource setting like Ghana, spoligotyping and MIRU-VNTR typing remain good alternatives.

TB in humans is caused mainly by *Mycobacterium tuberculosis* sensu stricto (MTBss) and *Mycobacterium africanum* (MAF), which are further divided into seven lineages: MTBss lineages 1–4 and 7 (L1–L4 and L7); MAF lineages 5 and 6 (L5 and L6) (Blouin et al., 2012, de Jong et al., 2010). While MTBss is distributed globally, MAF is restricted to West Africa, where it is responsible for up to 50% of TB cases (Gagneux and Small, 2007). Nevertheless, reports mainly from the Gambia where L6 is prevalent, suggest MAF is attenuated compared to MTBss, hence could be outcompeted by MTBss (de Jong et al., 2010, de Jong et al., 2008, Kallenius et al., 1999). However, an 8-year study recently conducted in Ghana found the prevalence of MAF to be fairly constant at approximately 20%, indicating that MAF and MTBss may be transmitted equally (Yeboah-Manu et al., 2016). The objective of this study was to determine the transmission dynamics of TB caused by MTBss and MAF in Ghana.

## Methods

### Study design and population

This study was a population-based prospective study in which sputum samples were collected from consecutive clinically diagnosed pulmonary TB patients reporting to 12 selected health facilities within an urban setting (Accra Metropolitan Assembly (AMA)) and the rural setting of East Mamprusi District (MamE) (Supplementary material, Figure S1). The study was conducted from July 2012 to December 2015. A pulmonary TB case was defined as an individual with a case of TB that was confirmed both clinically and bacteriologically. Detailed demographic and epidemiological data were obtained from consented participants.

### Mycobacterial isolation, species identification, and drug susceptibility testing

The sputum samples were decontaminated and cultured on Lowenstein–Jensen medium to obtain mycobacterial isolates. These isolates were confirmed as MTBC by detecting the MTBC-specific insertion sequence IS*6110* using PCR (Yeboah-Manu et al., 2001). In vitro drug susceptibility to isoniazid and rifampicin were determined using either the microplate Alamar Blue cell viability assay, as described elsewhere (Otchere et al., 2016), and/or the GenoType MTBDR*plus* assay (Hain Lifescience), following the manufacturer’s protocol (Barnard et al., 2008).

### Lineage and strain classification

Lineage and strain classification of the MTBC was achieved in a stepwise manner using large sequence polymorphism typing identifying regions of difference 4, 9, 12, 702, and 711 (de Jong et al., 2010, Gagneux and Small, 2007), single nucleotide polymorphism typing, spoligotyping (Kamerbeek et al., 1997), and MIRU-VNTR typing (Supply et al., 2006). For MIRU-VNTR typing, a customized set of 8 MIRU loci was first used, as described by Asante-Poku et al. (2014), and clustered cases were resolved by analyzing the remaining 7 loci of the standard MIRU-15 loci set (Supply et al., 2006). All assays were well controlled with PCR amplifications and pre-PCR procedures conducted in physically separated compartments to avoid laboratory cross-contamination. The presence of more than one allelic repeat number (multiple allele) for any given locus is suggestive of laboratory cross-contamination, multiple strain infection, or microevolution of a single strain. To prevent bias resulting from cross-contamination and multiple strain infection, isolates with multiple alleles at more than one MIRU locus (described as ‘untypeable’) were excluded from further analysis. Isolates with only one multiple allele at any given locus were, however, included due to the possibility of microevolution.

The spoligotyping patterns and assigned shared type numbers obtained were defined according to the SITVITWEB database (http://www.pasteur-guadeloupe.fr: 8081/SITVIT_ONLINE/), while sub-lineages were assigned based on the MIRU-VNTRplus database (http://www.miru-vntrplus.org) (Allix-Beguec et al., 2008). Strains with no lineage nomenclature data were further identified using the TB lineage database (Shabbeer et al., 2012) or otherwise regarded as orphan strains. A strain was defined as an MTBC isolate with a unique molecular signature, and thus a unique spoligotype pattern and/or a unique MIRU-VNTR allelic pattern for the number of investigated MIRU loci.

### Clustering analysis and risk factor assessment

Clustering analysis was performed using the categorical parameter and the unweighted pair group method with arithmetic mean (UPGMA) coefficient from a constructed phylogenetic tree using the online MIRU-VNTR tool. Clustering analysis was based on the assumption that strains with the same DNA fingerprint may be epidemiologically linked and associated with recent TB transmission (Hall, 1996). A cluster was defined as two or more isolates (same strain) that share an indistinguishable spoligotype and 15-locus MIRU-VNTR allelic pattern, but allowing for one missing allelic data at any one of the difficult-to-amplify MIRU loci (VNTR 2163, 3690, and 4156). The size of a cluster was also defined using the total number of isolates in the cluster classified into categories of small (2 isolates), medium (3–5 isolates), large (6–20 isolates), and very large (>20 isolates).

The recent transmission rate was estimated using the *n *− 1 formula (Glynn et al., 1999): $\frac{\left(nc-c\right)}{n}$, where *nc* is the total number of clustered cases, *c* is the number of clusters, and *n* is the total number of cases in the sample.

Only one strain per participant was included in the analysis, and follow-up cases were excluded. The clustering analysis was stratified first by location and then by MTBC lineage. The spatial distribution and clustering among all of the observed Spoligo/MIRU strain types were studied by constructing a minimum spanning tree (MST) with Bionumerics software (Applied Maths, Sint-Marteen-Latem, Belgium).

### Data management and analysis

Both molecular and epidemiological data were analyzed. Epidemiological data retrieved from all participants with positive MTBC cultures were included in the analysis while excluding data from those with no growth, contaminated cultures, and isolated non-tuberculous mycobacterial species. All statistical analyses were conducted using the Stata statistical package version 14.2 (Stata Corp., College Station, TX, USA). The association of specific lineages and/or sub-lineages of the MTBC with time and/or geographical locations were explored using the Chi-square test and a logistic regression model. For the determination of independent predictive factors for recent TB transmission, a multivariate analysis (forward stepwise approach with a probability entry of 0.1) was conducted using a logistic regression model while estimating the odds ratios (OR). *p*-Values of <0.05 were considered significant.

The study is reported according to the Strengthening the Reporting of Molecular Epidemiology for Infectious Diseases (STROME-ID) guidelines (Field et al., 2016).

## Results

### Characteristics of study participants

**Figure 1:**
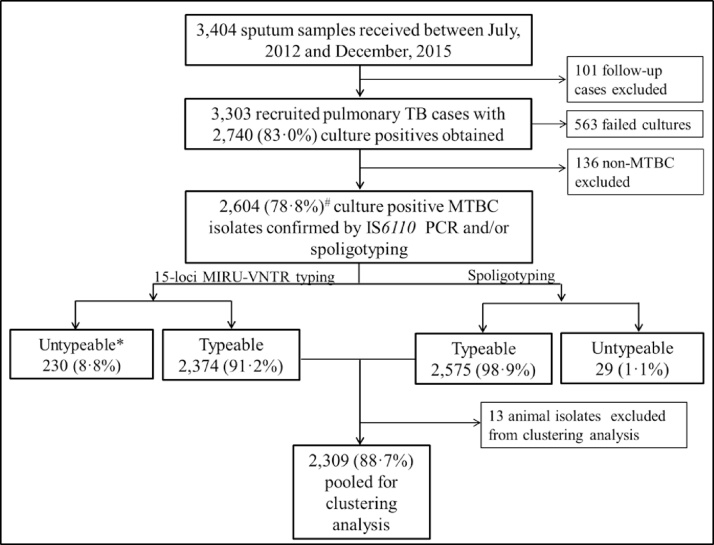
Pipeline for recruited participants and culture-positive TB cases included in the clustering analysis. *Category described as untypeable for MIRU-VNTR includes isolates with ≥2 MIRU loci unamplified (*n* = 164, 71.3%) and isolates with a double allele at ≥2 MIRU loci (*n* = 66, 28.7%). These isolates were described as suspected mixed infection or laboratory contamination and hence were excluded from further analysis. #Frequency was expressed as the total number of *Mycobacterium tuberculosis* complex (MTBC) isolates obtained.

A total 3303 sputum smear-positive pulmonary TB cases were recruited, 382 (11.6%) from the rural setting and 2921 (88.4%) from the urban setting; 2604 (78.8%) MTBC isolates were obtained from these cases (Supplementary material, Table S1). After excluding 13 *Mycobacterium bovis* and isolates that were untypeable (described in the Methods section), 2309 of 2604 isolates (88.7%) were included for clustering analysis. The participants comprised 1631 (71%) males and 663 (29%) females (there was no record of sex for 15 participants) with a median age of 39 years (range 3–91 years) and 33 years (range 4–90 years), respectively (Figure 1; Supplementary material, Table S1). The male-to-female ratio observed was comparable to the national average of approximately 2:1.

Of the 2309 participants with MTBC genotyping results, 201 (8.7%) were from the rural setting and 2108 (91.3%) from the urban setting. Among this study cohort, 7.4% (184/2482) of participants were previously treated cases including relapse, which is similar to the national value of 7.0% (WHO, 2015). Seventy-one percent (1561/2208) presented with a sputum smear microscopy bacterial burden result of at least 2+ and 33% (544/1665) admitted having contact with at least one TB patient. In a multivariate logistic regression analysis, it was found that male patients were less likely to be infected with a L5 strain (adjusted OR 0.7, 95% confidence interval (CI) 0.5–0.9) and individuals living in villages were more likely to be infected with a L6 strain (OR 6.6, 95% CI 1.2–36.1) (Supplementary material, Table S2).

### Population structure and recent transmission rate estimation

**Figure 2:**
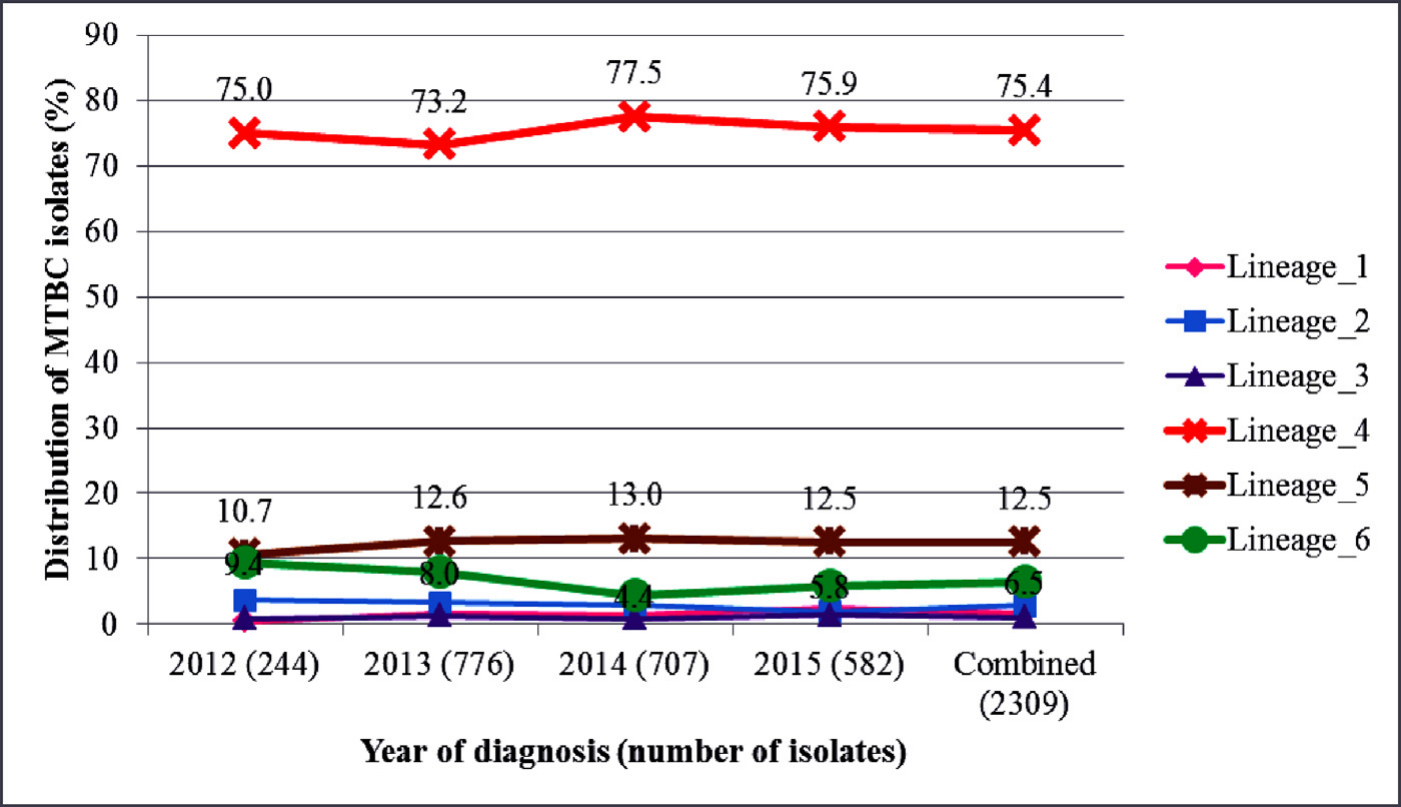
Temporal distribution of 2309 *Mycobacterium tuberculosis* complex (MTBC) isolates stratified by lineage. Lineages are color-coded with the universally accepted color codes for the main MTBC lineages.

Among the 2309 MTBC isolates analyzed for clustering, 1870 (81.0%) were MTBss and 439 (19.0%) were MAF. Six of the seven human-adapted MTBC lineages were found, with L4, L5, and L6 being most frequent: 1741 (75.4%), 289 (12.5%), and 150 (6.5%) isolates, respectively (Table 1). The relative proportions of the most frequent MTBC lineages remained constant over the entire 3.5-year study period (*p*_trend_: L4 *p* = 0.72, L5 *p* = 0.84, L6 *p* = 0.25; Figure 2).

**Table 1:** Geographical distribution and population structure of MTBC in Ghana by spoligotyping.

	Rural, *n* (%)	Urban, *n* (%)	Combined, *n* (%)^a^
MTBC isolates	204 (8.8)	2118 (91.2)	2322
Species distribution			
* M. tuberculosis*	172 (9.2)	1698 (90.8)	1870 (80.5)
* M. africanum*	29 (6.6)	410 (93.4)	439 (18.9)
Animal	3 (23.1)	10 (76.9)	13 (0.6)

Human adapted MTBC lineage distribution			
Lineage_1	4 (10.5)	34 (89.5)	38 (1.6)
Lineage_2	14 (21.5)	51 (78.5)	65 (2.8)
Lineage_3	1 (3.8)	25 (96.2)	26 (1.1)
Lineage_4	153 (8.8)	1588 (91.2)	1741 (75.4)
Lineage_5	15 (5.2)	274 (94.8)	289 (12.5)
Lineage_6	14 (9.3)	136 (90.7)	150 (6.5)

Lineage_4 sub-lineage distribution			
Cameroon	77 (7.4)	969 (92.6)	1046 (60.1)
Ghana	50 (13.3)	326 (86.7)	376 (21.6)
Haarlem	12 (7.7)	144 (92.3)	156 (9.0)
LAM	7 (14.0)	43 (86.0)	50 (2.9)
Uganda	1 (2.5)	39 (97.5)	40 (2.3)
Other (S, U, X, NEW-1)	5 (9.8)	46 (90.2)	51 (2.9)
Not determined	1 (4.5)	21 (95.5)	22 (1.3)

MTBC, *Mycobacterium tuberculosis* complex.

a Proportions stated here are column-wise distributions with respect to the categories of species, lineages or sub-lineages.

**Figure 3:**
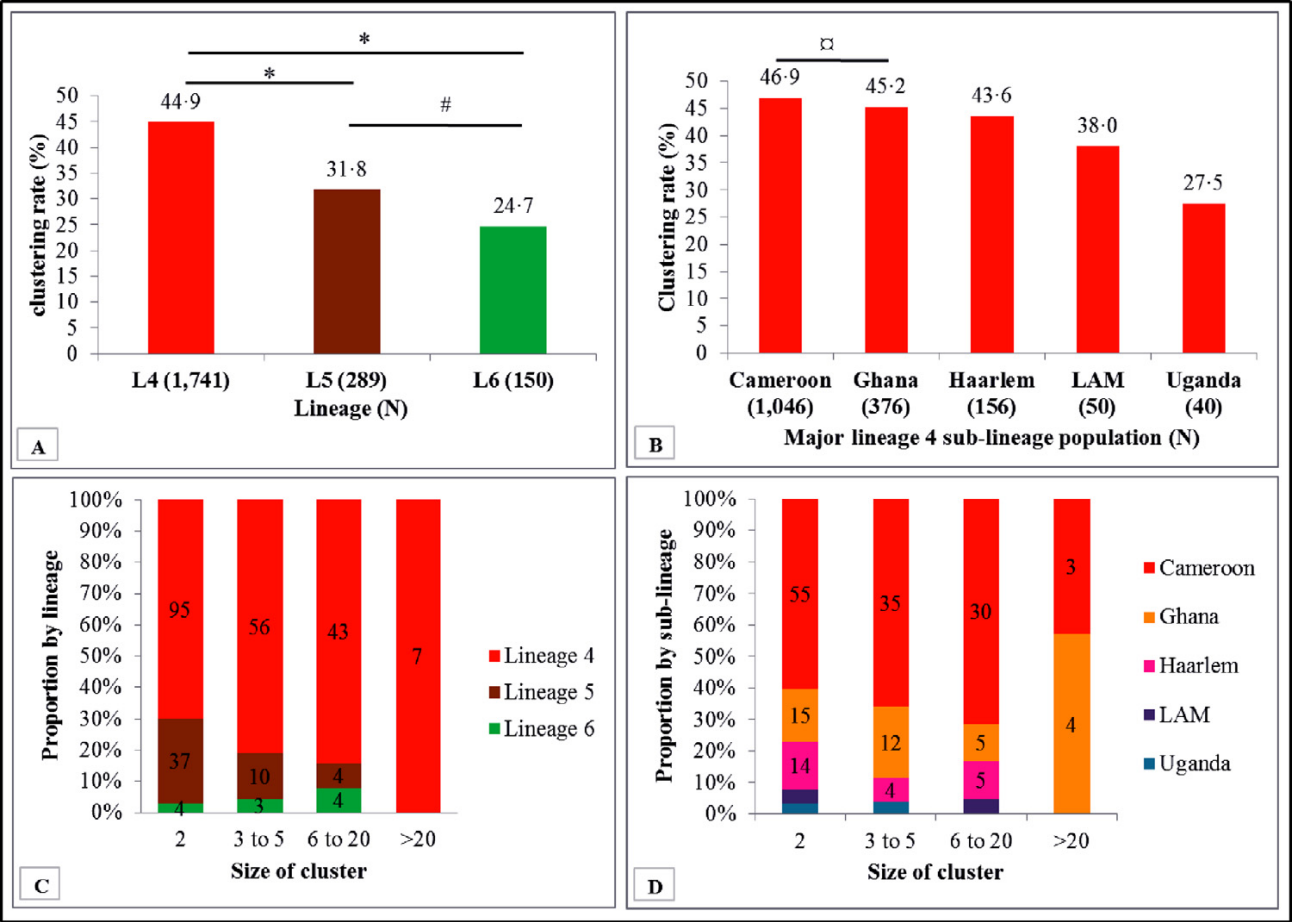
Cluster distribution and size stratified by lineage (panel A and C) and sub-lineage (panel B and D). *p < 0.001, #*p* = 0.118, ¤*p* = 0.565.

Of the 2309 isolates included for clustering analysis, 1227 (53.1%) isolates clustered in 276 different clusters with a mean cluster size of 4 (range 2–35) and 1082 (46.9%) unique isolates were identified, giving a total of at least 1358 different MTBC strains circulating within the study population (Table 2a). Using the *n* − 1 method, the overall clustering rate (reflecting the recent transmission rate) was estimated to be 41.2%. Lineages 2, 4, and 5 contributed high clustering rates of 53.8%, 44.9%, and 31.8%, respectively (Table 2a). The Cameroon, Ghana, and Haarlem sub-lineages of L4 were the most abundant sub-lineages and, compared to the LAM sub-lineage, contributed significantly to the observed high L4 clustering rate (*p <* 0.05) (Figure 3). There was no significant difference in the clustering rate between the Cameroon and Ghana sub-lineages (*p* = 0.57) (Figure 3). While no significant difference in the recent transmission rates was seen between members of MAF (L5 and L6, *p* = 0.118), it was found that L4 was transmitted significantly more (*p* < 0.001), with seven of its clusters having very large cluster sizes (>20 isolates per cluster) made up of the Ghana sub-lineage (four very large clusters) and Cameroon sub-lineage (three very large clusters) (Figure 3; Supplementary material, Figure S2). Notwithstanding the lower transmissibility of L5 and L6 compared to L4, four large clusters were also observed for each of these lineages. The urban and rural settings had estimated recent transmission rates of 41.7% and 9.0%, respectively.

**Table 2a:** Clustering analysis stratified by lineages and major sub-lineage populations of MTBC.

Lineage	Isolates (*n*)	Clustered cases (c)	Clustered strains (nc)	Single cases (s)	Total strain types (s + c)	Clustering rate^a^ (%)
Lineage 1	38	3	7	31	34	10.5
Lineage 2	65	8	43	22	30	53.8
Lineage 3	26	2	4	22	24	7.7
Lineage 4	1741	201	982	759	960	44.9
Cameroon^b^	1046	123	614	432	555	46.9
Ghana^b^	376	36	206	170	206	45.2
Haarlem^b^	156	23	91	65	88	43.6
LAM^b^	50	6	25	25	31	38.0
Uganda^b^	40	5	16	24	29	27.5
Lineage 5	289	51	143	146	197	31.8
Lineage 6	150	11	48	102	113	24.7
Summary^c^	2309	276	1227	1082	1358	41.2

MTBC, *Mycobacterium tuberculosis* complex.

a The clustering rate was used to estimate the recent transmission rate.

b Major lineage 4 sub-population.

c The summary was calculated using only the items in cells corresponding to the six main lineages.

### Exploring the diversity and clustering within the MTBC lineages

**Figure 4:**
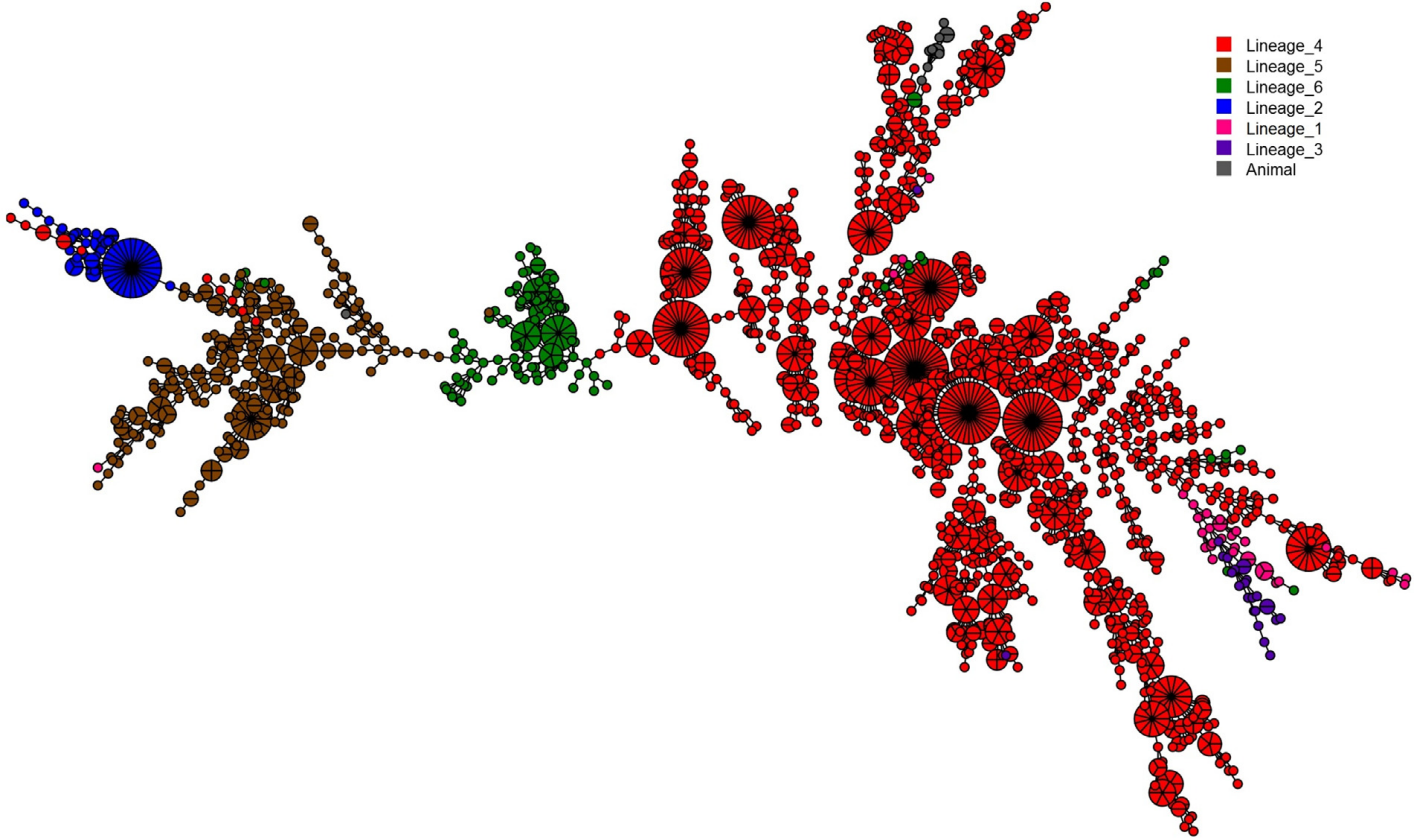
Minimum spanning tree (MST) representation of the clustering of 2322 *Mycobacterium tuberculosis* complex (MTBC) isolates from Accra Metropolitan Assembly and East Mamprusi District built with Bionumerics software. The color code reflects the main MTBC lineages 1 to 6 with the size depicting the number of clustered isolates with an identical strain type.

Very large molecular clusters (clusters with >20 isolates; defined in the Methods section) were observed for L4, in addition to one strikingly large cluster belonging to the Beijing family of lineage 2 (Figure 4; Supplementary material, Figure S3). Generally, only a few multidrug-resistant MTBC strains were observed across all the major lineages (Supplementary material, Figures S4–S6). There was no single large cluster with all isolates being multidrug-resistant (Supplementary material, Figure S4). The spatial distributions of the isolates constituting each cluster stratified by study setting are shown in the Supplementary material, Figures S7–S9.

### Molecular epidemiology and factors associated with clustering: logistic regression modeling

Risk factors associated with recent TB transmission were sought. A total of 675 individuals belonging to either large (6–20 isolates) or very large (>20 isolates) molecular clusters were identified, with a combined median cluster size of 14 (range 6–35). The majority of the individuals belonging to very large clusters were male, with a male-to-female ratio of approximately 3:1, significantly higher than the 2:1 ratio observed in the general TB patient population (*p* = 0.022). Three large clusters – cluster ID MSC4193, MSC5003.X, and MSC4107, with cluster sizes of 9, 7, and 7 respectively – involved only male subjects (Table 3).

**Table 2b:** Clustering analysis stratified by study setting and lineages/major sub-lineage populations of MTBC.

Lineage	Isolates (*n*)		Clustered cases (c)		Clustered strains (nc)		Single cases (s)		Total strain types (s + c)		Clustering rate^a^ (%)	
	Urban	Rural	Urban	Rural	Urban	Rural	Urban	Rural	Urban	Rural	Urban	Rural
Lineage 1	34	4	3	0	7	0	27	4	30	4	11.8	0
Lineage 2	51	14	5	1	33	4	18	10	23	11	54.9	21.4
Lineage 3	25	1	2	0	4	0	21	1	23	1	8	0
Lineage 4	1588	153	183	10	907	25	681	128	864	138	45.6	9.8
Cameroon^b^	969	77	112	5	575	10	394	67	506	72	47.8	6.5
Ghana^b^	326	50	32	4	182	12	144	38	176	42	46	16
Haarlem^b^	144	12	20	1	81	3	63	9	83	10	42.4	16.7
LAM^b^	43	7	6	0	25	0	18	7	24	7	44.2	0
Uganda^b^	39	1	5	0	16	0	23	1	28	1	28.2	0
Lineage 5	274	15	49	0	137	0	137	15	186	15	32.1	0
Lineage 6	136	14	10	0	43	0	93	14	103	14	24.3	0
Summary^c^	2108	201	252	11	1131	29	977	172	1229	183	41.7	9

MTBC, *Mycobacterium tuberculosis* complex.

a The clustering rate was used to estimate the recent transmission rate.

b Major lineage 4 sub-population.

c The summary was calculated using only the items in cells corresponding to the six main lineages.

**Table 3:** Characteristics of large molecular clusters resulting from combined 15-MIRU and spoligotyping cluster analysis.

Number	Cluster code^a^	Number of cases in cluster	Sex, male: female	Median age (IQR)	Diagnosis lapse^b^ (months)	Same residential district^c^	Known risk factor (number)^d^	Lineage (sub-lineage)	Drug resistance^e^
**1**	**MSC4063.X**	35	31:4	34 (26–44)	40	7/5/5/4/4/5	Smoking (6) Other (8)	L4 (Cameroon)	3
2	MSC4060.X	34	24:10	34 (25–45)	41	6/4/3/3/3/3	Smoking (6) Other (5)	L4 (Cameroon)	4
3	MSC4045.X	30	26:4	40 (29–48)	39	7/3/3/3/3	Smoking (5) HIV (4) Other (3)	L4 (Cameroon)	2
4	**MSC2001**	27	22:5	35 (27–48)	37	8/5	Smoking (8) HIV (4) Other (2)	L2 (Beijing)	1
5	MSC4031	26	19:7	41 (33–52)	36	6/4/3	Smoking (6) HIV (3) Other (1)	L4 (Ghana)	11
6	MSC4110	26	21:5	38 (28–51)	39	6/3	Smoking (5) HIV (1) Other (4)	L4 (Ghana)	ND
7	**MSC4095**	24	16:8	35 (24–45)	39	7/6	Smoking (5) Other (2)	L4 (Ghana)	9
8	MSC4027	21	16:5	27 (25–45)	40	6/3	Smoking (3)	L4 (Ghana)	3
9	MSC4063.3	19	18:1	28 (21–45)	41	5/5	Smoking (7)	L4 (Cameroon)	ND
10	**MSC4063.18**	18	10:7	35 (24–41)	36	6/3	Smoking (4) HIV (1)	L4 (Cameroon)	ND
11	MSC4013	15	13:2	42 (32–55)	32	4/3	Smoking (3) HIV (2) Other (2)	L4 (Haarlem)	2
12	MSC4136	15	13:2	36 (28–44)	34	6	Smoking (2) HIV (1) Other (3)	L4 (Haarlem)	ND
13	MSC4040	14	8:6	31 (27–45)	33	3/3	HIV (1) Other (4)	L4 (Cameroon)	1
14	**MSC4069.X**	14	11:3	27 (23–38)	34	6/3	Smoking (2) HIV (2) Other (1)	L4 (Cameroon)	ND
15	MSC4073	14	9:5	40 (29–47)	24	5/4	Smoking (3)	L4 (Cameroon)	3
16	MSC5002.X	14	7:7	40 (38–53)	28	5	HIV (2) Smoking (1)	L5 (West African I)	2
17	MSC4063.2	13	8:4	37 (27–44)	38	4	Smoking (2) Other (3)	L4 (Cameroon)	ND
18	MSC4068.X	13	9:4	35 (30–44)	27	5/3	Smoking (6) HIV (2) Other (2)	L4 (Cameroon)	ND
19	MSC4024	12	6:5	28 (26–42)	37	3	Smoking (2) Other (1)	L4 (X3)	4
20	MSC4060.18	12	7:5	35 (32–40)	36	3/3	Smoking (2) HIV (2) Other (3)	L4 (Cameroon)	1
21	MSC4063.17	12	7:5	26 (24–51)	39	ND	Smoking (4) HIV (1) Other (3)	L4 (Cameroon)	2
22	MSC4138	11	7:4	41 (30–48)	28	4	Smoking (4)	L4 (LAM)	ND
23	MSC4069.3	10	5:5	32 (24–39)	31	3	Smoking (1) HIV (1)	L4 (Cameroon)	2
24	**MSC4104**	10	7:2	35 (25–54)	34	5	Smoking (3) Other (1)	L4 (Ghana)	6
25	MSC6006	10	4:6	41 (35–47)	33	5/3	Smoking (1) Other (2)	L6 (West African II)	ND
26	MSC4045.3	9	7:2	43 (32–50)	33	3	Smoking (1) Other (1)	L4 (Cameroon)	1
27	MSC4060.21	9	6:3	32 (26–43)	22	ND	HIV (1) Other (2)	L4 (Cameroon)	ND
28	MSC4060.3	9	5:4	32 (25–53)	34	3	Smoking (1) Other (2)	L4 (Cameroon)	ND
29	MSC4193	9	9:0	36 (30–41)	28	ND	Smoking (6) HIV (1) Other (2)	L4 (Cameroon)	ND
30	MSC4068.3	8	6:2	45 (34–54)	27	2	Smoking (2) HIV (1) Other (1)	L4 (Cameroon)	ND
31	MSC4022	7	6:1	50 (46–62)	36	4	Smoking (1) Other (1)	L4 (Haarlem)	ND
32	MSC4060.4	7	3:4	34 (30–49)	22	5	Smoking (1)	L4 (Cameroon)	ND
33	MSC4080.13	7	4:3	24 (17–50)	20	3	Smoking (1)	L4 (Cameroon)	1
34	MSC4082	7	6:1	35 (28–40)	33	3	Smoking (1)	L4 (Ghana)	ND
35	MSC4107	7	7:0	38 (29–53)	28	3/3	Smoking (2) Other (1)	L4 (Ghana)	1
36	MSC5003.2	7	4:3	35 (26–57)	33	ND	HIV (1)	L5 (West African I)	1
37	MSC5003.X	7	7:0	43 (26–66)	34	3	Smoking (2) Other (1)	L5 (West African I)	ND
38	MSC6004	7	5:2	44 (36–50)	31	ND	HIV (1) Other (3)	L6 (West African II)	3

MIRU, mycobacterial interspersed repetitive unit; L2, lineage 2; L4, lineage 4; L5, lineage 5; L6, lineage 6; ND, none determined; IQR, interquartile range.

a Cluster codes in bold font involved evidence of household transmission.

b Time lapse (in months) between first diagnosed case and last diagnosed case.

c Number of participants with the same district of residence. Only >2 individuals in the same residential district are indicated. ‘/’ is used to separate individuals from different districts.

d ‘Other’ in this category refers to alcohol or substance abuse.

e Number of participants carrying strains with drug resistance to either isoniazid or rifampicin.

Epidemiological investigations revealed both localized and dispersed recent transmission among the clustered cases, with suggested evidence of household transmission in at least six large clusters (MSC4063.X, MSC2001, MSC4095, MSC4063.18, MSC4069.X, and MSC4104). Specifically, the same L4 strain (part of cluster MSC4069.X) was found among three individuals belonging to the same household, with the oldest person (age 49 years) reporting having contact with his son who had TB 4 months prior to his episode (suggestive of household transmission). The majority of the large clusters involved TB strains circulating over almost the entire study period (Supplementary material, Figure S10). Apart from three Ghana sub-lineage clusters (MSC4104, MSC4031, and MSC4095) and one L6 cluster (MSC6004), with respectively 60% (6/10), 42% (11/26), 38% (9/24), and 43% (3/7) of isolates showing resistance to rifampicin and/or isoniazid (Table 3), such high levels of drug resistance were not observed in the other large and very large clusters. Only 2% of the isolates belonging to large and very large clusters were multidrug-resistant TB strains and this was significantly lower than that for small (2 isolates) and medium (3–5 isolates) (4%) clusters (*p* = 0.031).

For the determination of possible factors associated with recent TB transmission, a general logistic regression model including all MTBC lineages was first performed, using the event of belonging to a clustered case as the outcome variable and participant variables as possible predictors (Table 4). In a separate logistic regression model, risk factors associated with recent TB transmission were tested stratified independently by L4 and L5 (Table 5), excluding L6 due to the limited sample size. In the multivariable analysis for the general logistic regression model, it was found that harboring either an isoniazid- or rifampicin-resistant MTBC strain (adjusted OR 0.7, 95% CI 0.5–0.9) was associated with a lower odds of belonging to a clustered case (Table 4). All other factors such as education status, occupation, income level, ethnicity, religion, and HIV status had no association with recent TB transmission.

**Table 4:** Logistic regression analysis of risk factors associated with TB clustering (recent TB transmission).

Variable	MTBC (*N* = 2309)		Univariate		Multivariate^a^	
	Total TB cases, *n* (%)	Clustered cases^b^, *n* (%)	OR (95% CI)	*p*-Value	Adjusted OR (95% CI)	*p*-Value
Year diagnosed	2309 (100)	1229 (53·2)				
2012	244 (10·6)	147 (60·3)	1·4 (1·0–1·8)	0·043	1·3 (0·9–1·7)	0·113
2013	776 (33·6)	410 (52·8)	Reference			
2014	707 (30·6)	365 (51·6)	1·0 (0·8–1·2)	0·642	0·9 (0·7–1·1)	0·203
2015	582 (25·2)	307(52·8)	1·0 (0·8–1·2)	0·975	1·0 (0·8–1·2)	0·703

Sex	2294 (99·4)					
Male	1631 (71·1)	863 (52·9)	1·0 (0·8–1·2)	0·685		
Female	663 (28·9)	357 (53·8)	Reference			

Age (years)^c^	2224 (96·3)					
<15	37 (1·7)	25 (67·6)	1·6 (0·8–3·3)	0·183	1·6 (0·8–3·2)	0·221
15–29	639 (28·7)	360 (56·3)	Reference			
30–39	570 (25·6)	307 (53·9)	0·9 (0·7–1·1)	0·387	0·9 (0·7–1·2)	0·688
40–59	778 (35·0)	398 (51·2)	0·8 (0·7–1·0)	0·052	0·9 (0·7–1·1)	0·241
>59	200 (9·0)	97 (48·5)	0·7 (0·5–1·0)	0·053	0·9 (0·6–1·1)	0·211

Nationality	1781 (77·1)					
Ghanaian	1714 (96·2)	932 (54·4)	Reference			
Other	67 (3·8)	38 (56·7)	1·1 (0·7–1·8)	0·706		
Locality	2309 (100)	1229 (53·2)				
Rural	201 (8·7)	74 (36·8)	Reference			
Urban	2108 (91·3)	1155 (54·8)	2·1 (1·5–2·8)	<0·001		

Residence classification	1642 (71·1)					
Village	69 (4·2)	27 (39·1)	0·5 (0·3–0·8)	0·007		
Town	182 (11·1)	96 (52·7)	0·9 (0·6–1·2)	0·415		

City residential area	52 (3·2)	27 (51·9)	0·8 (0·5–1·5)	0·564		
City suburb	1136 (69·2)	636 (56·0)	Reference			
City slum	203 (12·4)	112 (55·2)	1·0 (0·7–1·3)	0·83		

Residential district	1538 (66·6)					
Ablekuma	545 (35·4)	298 (54·7)	Reference			
Ashiedu Keteke	170 (11·1)	100 (58·8)	1·2 (0·8–1·7)	0·343		
Ayawaso	220 (14·3)	124 (56·4)	1·1 (0·8 to 1·5)	0·672		
Kpeshie	224(14·6)	121 (54·0)	1·0 (0·7–1·3)	0·867		
Mamprusi East	70 (4·6)	22 (31·4)	0·4 (0·2–0·6)	<0·001		
Okaikoi	176 (11·4)	98 (55·7)	1·0 (0·7 to 1·5)	0·816		
Osu Klottey	133 (8·6)	78 (58·7)	1·2 (0·8–1·7)	0·409		

Household type	1624 (70·3)					
Self-contained	412 (25·4)	221 (53·6)	1·0 (0·8–1·2)	0·797		
Compound house	1212 (74·6)	659 (54·4)	Reference			

Education	1748 (75·7)					
Primary	222 (12·7)	125 (56·3)	1·1 (0·8–1·5)	0·637		
Middle/JHS	637 (36·4)	347 (54·5)	Reference			
Secondary	429 (24·5)	232 (54·1)	1·0 (0·8–1·3)	0·899		
Tertiary	190 (10·9)	110 (57·9)	1·1 (0·8–1·6)	0·405		
No education	270 (15·4)	141 (52·2)	0·9 (0·7–1·2)	0·534		

Occupation	1722 (74·6)					
Unemployed	390 (22·6)	208 (53·3)	0·9 (0·7–1·1)	0·423		
Unskilled	951 (55·2)	530 (55·7)	Reference			
Skilled	381 (22·1)	198 (52·0)	0·9 (0·7–1·1)	0·213		

Monthly income (GH¢)	1622 (70·2)					
None	371 (22·9)	213 (57·4)	Reference			
<301	807 (49·7)	438 (54·3)	0·9 (0·7–1·1)	0·315		
301–1000	407 (25·1)	218 (53·6)	0·8 (0·6–1·1)	0·281		
>1000	37 (2·3)	15 (40·5)	0·5 (0·3–1·0)	0·052		

Religion	1771 (76·7)					
Christian	1361 (76·9)	739 (54·3)	Reference			
Islam	302 (17·0)	161 (53·3)	1·0 (0·7–1·2)	0·755		
Other	26 (1·5)	14 (53·9)	1·0 (0·4–2·1)	0·963		
Not religious	82 (4·6)	49 (59·7)	1·2 (0·8–2·0	0·366		

Ethnicity	1760 (76·4)					
Akan	570 (32·3)	309 (54·2)	Reference			
Ewe	259 (14·7)	143 (55·2)	1·0 (0·8–1·4)	0·788		
Ga/Adangbe	544 (30·8)	310 (57·0)	1·1 (0·9–1·4)	0·352		
Other	392 (22·2)	196 (50·0)	0·8 (0·6–1·1)	0·199		

Marital status	1758 (76·1)					
Single	766 (43·6)	431 (56·3)	Reference			
Married	742 (42·2)	395 (53·2)	0·9 (0·7–1·1)	0·237		
Divorced	167 (9·5)	99 (59·3)	1·1 (0·8–1·6)	0·476		
Widowed	83 (4·7)	35 (42·2)	0·6 (0·3–0·9)	0·015		

Smear positivity	2208 (95·6)					
Scanty 1–9	173 (7·8)	96 (55·5)	1·1 (0·8–1·5)	0·714		
1+	474 (21·5)	237 (50·0)	0·9 (0·7–1·1)	0·151		
2+	546 (24·7)	294 (53·9)	1·0 (0·8–1·2)	0·957		
3+	1015 (46·0)	548 (54·0)	Reference			

Previous TB treatment	1737 (75·2)					
Yes	291 (16·8)	153 (52·6)	0·9 (0·7–1·2)	0·535		
No	1446 (83·2)	789 (54·6)	Reference			

Risk of TB contact						
Close friend/household	1665 (72·1)					
No contact	1121 (67·3)	594 (53·0)	Reference			
1 contact	212 (12·7)	118 (55·7)	1·1 (0·8–1·5)	0·475		
2–5 contacts	309 (18·6)	179 (57·9)	1·2 (0·9–1·6)	0·123		
6–10 contacts	23 (1·4)	15 (65·2)	1·7 (0·7–4·0)	0·249		

Imprisonment	1660 (71·9)					
Yes	97 (5·8)	56 (57·7)	1·1 (0·8–1·7)	0·513		
No	1563 (94·2)	849 (54·3)	Reference			

Health/laboratory worker	1661 (71·9)					
Yes	47 (2·8)	25 (53·2)	0·9 (0·5–1·7)	0·85		
No	1614 (97·2)	881 (54·6)	Reference			

Immunosuppressive condition	1695 (73·4)					
Any	893 (52·7)	488 (54·6)	1·0 (0·9–1·2)	0·747		
None	802 (47·3)	432 (53·9)	Reference			

Diabetes mellitus	534 (23·1)					
Yes	104 (19·5)	54 (51·9)	1·0 (0·7–1·5)	0·957		
No	430 (80·5)	222 (51·6)	Reference			

HIV status	1166 (50·5)					
Positive	144 (12·3)	82 (56·9)	1·1 (0·8–1·6)	0·481		
Negative	1022 (87·7)	550 (53·8)	Reference			

Smoking	1518 (65·7)					
Yes	434 (28·6)	237 (54·6)	1·0 (0·8–1·2)	0·949		
No	1084 (71·4)	590 (54·4)	Reference			

Substance abuse (excluding alcohol)	1401 (60·7)					
Yes	140 (10·0)	84 (60·0)	1·3 (0·9–1·8)	0·172		
No	1261 (90·0)	680 (53·9)	Reference			

Substance abuse (including alcohol)	1474 (63·8)					
Yes	460 (31·2)	250 (54·3)	1·0 (0·8–1·3)	0·858		
No	1014 (68·8)	546 (53·8)	Reference			

Lineage	2309 (100)					
Lineage 1	38 (1·7)	7 (18·4)	0·2 (0·08–0·4)	<0·001	0·13 (0·05–0·36)	<0·001
Lineage 2	65 (2·8)	43 (66·2)	1·5 (0·9–2·5)	0·126	1·5 (0·9–2·5)	0·155
Lineage 3	26 (1·1)	4 (15·4)	0·1 (0·05–0·4)	<0·001	0·15 (0·05–0·45)	0·001
Lineage 4	1741 (75·4)	984 (56·5)	Reference			
Lineage 5	289 (12·5)	143 (49·5)	0·8 (0·6–1·0)	0·026	0·7 (0·6–0·9)	0·032
Lineage 6	150 (6·5)	48 (32·0)	0·4 (0·3–0·5)	<0·001	0·3 (0·2–0·5)	<0·001

Lineage 4 sub-lineage						
Cameroon	1046 (60·1)	616 (58·9)	Reference			
Ghana	376 (21·6)	206 (54·8)	0·8 (0·7–1·1)	0·167		
Haarlem	156 (9·0)	91(58·3)	1·0 (0·7–1·4)	0·895		
LAM	50 (2·9)	25 (50·0)	0·7 (0·4–1·2)	0·215		
Uganda	40 (2·3)	16 (40·0)	0·5 (0·2–0·9)	0·02		
Other	51 (2·9)	26 (51·0)	0·7 (0·4–1·3)	0·265		
Not determined	22 (1·3)	4 (18·2)	0·2 (0·1–0·5)	0·001		

Drug resistance	2300 (99·6)					
Any	313 (13·6)	138 (44·1)	0·6 (0·5–0·8)	<0·001	0·7 (0·5–0·9)	0·002
None	1987 (86·4)	1090 (54·9)	Reference			

Isoniazid mono-resistant	2300 (99·6)					
Yes	295 (12·8)	129 (43·7)	0·6 (0·5–0·8)	<0·001		
No	2005 (87·2)	1099 (54·8)	Reference			

Multidrug resistant (MDR)	2300 (99·6)					
Yes	81 (3·5)	35 (43·2)	0·7 (0·4–1·0)	0·063		
No	2219 (96·5)	1193 (53·8)	Reference			

Cluster size (*n*)	1227 (53·1)					
Small (2)	290 (23·6)					
Medium (3–5)	262 (21·4)					
Large (6–20)	452 (36·8)					
Very large (>20)	223 (18·2)					

MTBC, *Mycobacterium tuberculosis* complex; TB, tuberculosis; OR, odds ratio; CI, confidence interval; JHS, junior high school; GH¢, Ghanaian cedi.

a For the multivariate model, only variables with *p <* 0.1 and with at least 90% of available data were included. However ‘locality’ was excluded due to the small sample size from the rural setting. Residence classification, marital status, isoniazid mono-resistance, and MDR were excluded due to collinearity with other variables in the model.

b A cluster was defined as two or more isolates (same strain) that share an indistinguishable spoligotype and 15-locus MIRU-VNTR allelic pattern, but allowing for one missing allelic data at any one of the difficult-to-amplify MIRU loci.

c A significant decreasing trend in the probability of belonging to a clustered case was found with increasing age category (*p* = 0.004).

**Table 5:** Risk factors associated with TB clustering: logistic regression analysis stratified by lineage.^a^

Variables	Lineage 4 (*n* = 1741)		Univariate	Multivariate^b^		Lineage 5 (*n* = 289)		Univariate	
	TB cases, *n* (%)	Clustered cases^c^, *n* (%)	OR (95% CI)	Adjusted OR (95% CI)	*p*-Value	TB cases, *n* (%)	Clustered cases^c^, *n* (%)	OR (95% CI)	*p*-Value
Year diagnosed	1741 (100)					289 (100)			
2012	183 (10·5)	120 (65·6)	1·5 (1·1–2·1)*	1·4 (1·0–2·1)	0·062	26 (9·0)	14 (53·8)	1·2 (0·5–2·9)	0·659
2013	568 (32·6)	318 (56·0)	Reference			98 (33·9)	48 (49·0)	Reference	
2014	548 (31·5)	300 (54·7)	1·0 (0·8–1·2)	1·0 (0·7–1·3)	0·847	92 (31·8)	43 (46·7)	0·9 (0·5–1·6)	0·757
2015	442 (25·4)	244 (55·2)	1·0 (0·8–1·2)	1·0 (0·7–1·3)	0·955	73 (25·3)	38 (52·1)	1·2 (0·6–2·1)	0·691

Age (years)	1672					283			
<15	27 (1·6)	20 (74·1)	2·1 (0·9–5·0)			5 (1·8)	3 (60·0)		
15–29	497 (29·7)	289 (58·2)	Reference			78 (27·6)	42 (53·8)		
30–39	432 (25·8)	252 (58·3)	1·0 (0·8–1·3)			68 (24·0)	31 (45·6)		
40–59	580 (34·7)	315 (54·3)	0·9 (0·7–1·1)			94 (33·2)	48 (51·1)		
>59	136 (8·1)	71 (52·2)	0·8 (0·5–1·2)			38 (13·4)	16 (42·1)		

Locality	1741 (100)					289 (100)			
Rural	153 (8·8)	59 (38·6)	Reference			15 (5·2)	4 (26·7)	Reference	
Urban	1588 (91·2)	923 (58·1)	2·2 (1·6–3·1)**			274 (94·8)	139 (50·7)	2·8 (0·9–9·1)	0·081

Residential district	1165					189			
Ablekuma	412 (35·4)	237 (57·5)	Reference			77 (40·7)	39 (50·7)	Reference	
Ashiedu Keteke	132 (11·3)	81 (61·4)	1·2 (0·8–1·8)			13 (6·9)	5 (38·5)	0·6 (0·2–2·0)	0·419
Ayawaso	178 (15·3)	111 (62·4)	1·2 (0·8–1·8)			21 (11·1)	7 (33·3)	0·5 (0·2–1·4)	0·163
Kpeshie	166 (14·2)	88 (53·0)	0·8 (0·6–1·2)			37 (19·6)	25 (67·6)	2·0 (0·9–4·6)	0·091
Mamprusi East	56 (4·8)	19 (33·9)	0·4 (0·2–0·7)*			4 (2·1)	1 (25·0)	0·32 (0·03–3·26)	0·339
Okaikoi	134 (11·5)	80 (59·7)	1·1 (0·7–1·6)			24 (12·7)	12 (50·0)	1·0 (0·4–2·4)	0·956
Osu Klottey	87 (7·5)	58 (66·7)	1·5 (0·9–2·4)			13 (6·9)	5 (38·5)	0·6 (0·2–2·0)	0·419

Monthly income (GH¢)	1222								
None	275 (22·5)	167 (60·7)	Reference						
<301	605 (49·5)	351 (58·0)	0·9 (0·7–1·2)						
301–1000	314 (25·7)	184 (58·6)	0·9 (0·7–1·3)						
>1000	28 (2·3)	11 (39·3)	0·4 (0·2–0·9)*						

Marital status	1322								
Single	591 (44·7)	355 (60·1)	Reference						
Married	549 (41·5)	312 (56·8)	0·9 (0·7–1·1)	0·9 (0·7–1·2)	0·589				
Divorced	124 (9·4)	78 (62·9)	1·1 (0·8–1·7)	1·1 (0·7–1·7)	0·543				
Widowed	58 (4·4)	24 (41·4)	0·5 (0·3–0·8)*	0·5 (0·3–0·8)	0·011				

Lineage 4 sub-lineage									
Cameroon	1046 (60·1)	614 (58·7)							
Ghana	376 (21·6)	206 (54·8)	0·9 (0·7–1·1)	0·9 (0·7–1·2)	0·403				
Haarlem	156 (9·0)	91 (58·3)	1·0 (0·7–1·4)	1·0 (0·7–1·5)	0·87				
LAM	50 (2·9)	25 (50·0)	0·7 (0·4–1·2)	0·7 (0·4–1·4)	0·354				
Uganda	40 (2·3)	16 (40·0)	0·5 (0·2–0·9)*	0·4 (0·2–0·8)	0·013				
Other	51 (2·9)	26 (51·0)	0·7 (0·4–1·3)	0·8 (0·4–1·6)	0·558				
Not determined	22 (1·3)	4 (18·2)	0·2 (0·1–0·5)*	0·10 (0·03–0·35)	<0·001				

Drug resistance	1736								
Any	241 (13·9)	114 (47·3)	0·7 (0·5–0·9)*	0·7 (0·5–1·0)	0·059				
None	1495 (86·1)	867 (58·0)	Reference						

TB, tuberculosis; OR, odds ratio; CI, confidence interval; GH¢, Ghanaian cedi.

a Only variables with *p <* 0.1 from the general logistic regression model in Table 4 were included in this analysis. *p < 0.05; **p < 0.001.

b For the multivariate model, only variables with *p <* 0.1 and with at least 90% of available data were included.

c A cluster was defined as two or more isolates (same strain) that share an indistinguishable spoligotype and 15-locus MIRU-VNTR allelic pattern, but allowing for one missing allelic data at any one of the difficult-to-amplify MIRU loci.

**Figure 5:**
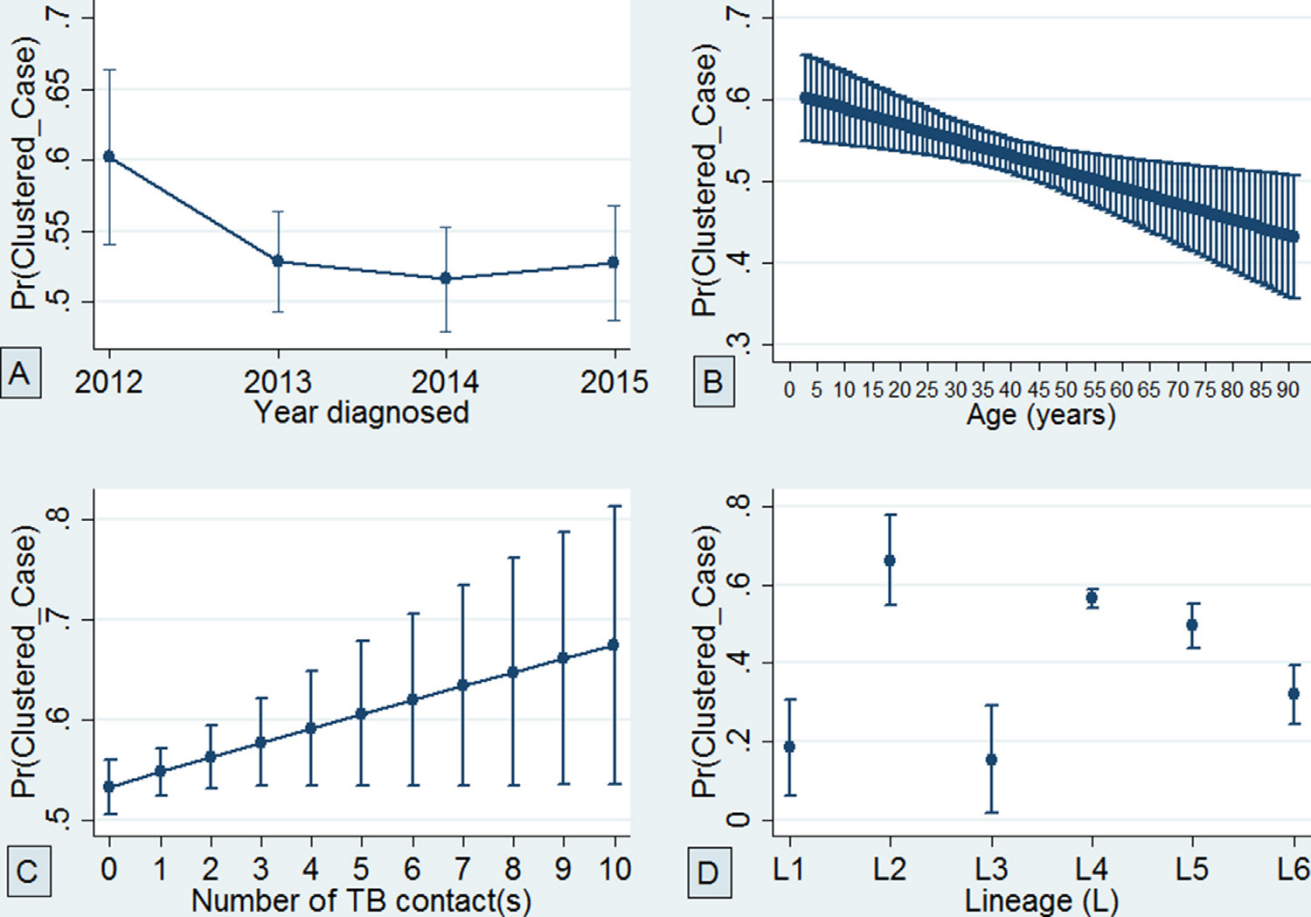
Adjusted predictions of the probability of belonging to a clustered case with 95% confidence interval: (A) at the year of diagnosis, (B) while ageing, (C) considering the number of close TB contact(s), and (D) considering the number of circulating *Mycobacterium tuberculosis* complex (MTBC) lineages.

Finally, using adjusted predictions, it was found that the probability of belonging to a clustered case decreased with age and increased with the number of TB contacts (Figure 5). In a separate logistic regression analysis, including age as a continuous variable with belonging to a clustered case as the outcome variable, it was found that each year increase in age was significantly associated with an approximately 1% (95% CI 0.13–2.00%) decrease in the odds of a TB patient being part of a recent transmission event (*p* = 0.007).

## Discussion

The aims of this study were to conduct a population-based prospective molecular epidemiological study to analyze the transmission dynamics of MTBC strains circulating in Ghana and to identify risk factors associated with recent TB transmission.

A high MTBC isolate recovery rate of 78.8% was obtained, higher than that reported in similar studies (Hamblion et al., 2016, Mears et al., 2015) and this strengthens the power of the sample size to make assessments of the TB transmission rate in Ghana. This study identified a high TB clustering (recent TB transmission) rate of 41.2%, which is quite alarming, with the urban and rural areas having estimated rates of 41.7% and 9.0%, respectively (Table 2b). These findings call for intensifying community outreach programs to encourage early case reporting and infection control. Moreover, the analysis predicted the probability of clustering to generally increase with the increase in the number of TB contacts (Figure 5). This means that a susceptible individual is likely to have TB and be involved in a recently transmitted event as the number of TB contacts increases.

Within the study population, no association of recent TB transmission was found with education status, occupation, income level, ethnicity, religion, or HIV status. However, it was observed that individuals below the age of 30 years were associated with recent TB transmission, and this is similar to observations made elsewhere (Hamblion et al., 2016, Vluggen et al., 2017). Also in this study, it was observed that each year increase in age was associated with an approximately 1% (95% CI 0.13–2.00; *p* = 0.007) decrease in the odds of a TB patient being part of a recent transmission event, implying that compared to younger individuals, older individuals are more likely to get active TB disease by reactivation of latent TB infection rather than through a recent transmission event (Hamblion et al., 2016). This finding puts age as a risk factor for recent TB transmission in Ghana. However, this finding was largely driven by L4 and L5, since separate analysis was not valid for L6 due to the small sample size. Furthermore, it was found that the male-to-female ratio among very large clusters was significantly higher than that observed in the general TB patient population (*p* = 0.022). This finding, together with the observation that some large clusters involved only male subjects, also indicates that males have a higher risk of recent TB transmission compared to females, suggesting that males may engage in certain social activities that predispose them to belonging to a recent transmission event.

A lower rate of multidrug-resistant TB was seen among large clustered cases compared to the general population (2% vs. 4%, *p* = 0.031), indicating a low multidrug-resistant TB transmissibility within the study population. This finding further suggests that the majority of drug-resistant TB cases in Ghana acquired the drug resistance during treatment, which indicates poor patient compliance (Danso et al., 2015). Moreover, it was also found that compared to drug (isoniazid and/or rifampicin)-sensitive MTBC strains, it was unlikely to find MTBC strains with isoniazid and/or rifampicin resistance involved in a recent transmission event (adjusted OR 0.7, 95% CI 0.5–0.9).

Within the study setting, a reduced transmission of MAF (L5: 31.8%, L6: 24.7%) compared to MTBss L4 (44.9%) was observed. The high recent transmission rate observed for L4 was driven by both the Cameroon and Ghana sub-lineages, with no difference in their transmissibility, hence identifying these sub-lineages as very important pathogens. The high recent transmission of the Ghana sub-lineage coupled with recently reported association with drug resistance (Otchere et al., 2016) is of public health importance and hence calls for the national tuberculosis control program to support peripheral diagnostic laboratories with facilities to accurately detect and help control the spread of the Ghana sub-lineage.

The higher recent transmission rate for L4 compared to L5 and L6 may not necessarily imply the outcompeting of L5 and L6 by L4, as their relative proportions remained constant over the entire study period (Figure 2) and also based on previous reports (Yeboah-Manu et al., 2016). Despite the low transmissibility of MAF, the observed stable relative proportion over the entire study period may be because the pathogen has adapted to infecting specific host populations (possibly due to unidentified host genetic or environmental factors peculiar to some West African inhabitants), hence enabling the maintenance of a stable prevalence over time. Using adjusted predictions for the probability of clustering, it was found that MAF L5 may still have the propensity to transmit equally to lineage 4 (Figure 5), not forgetting the confounding effect of a higher diversity in spoligotype pattern of L5 compared to L4 and hence reduced clustering of the former (Asante-Poku et al., 2016). Compared to L4, a significant association of L6 with individuals living in villages was found (OR 6.6, *p <* 0.05; Supplementary material, Table S2). The low recent TB transmission in the villages coupled with an association of L6 could be the reason why low frequencies of L6 strains were observed within the study setting.

This report could be limited by the possibility of an underestimation of the recent transmission rate resulting from the misclassification of strains as unique if they were actually clustered outside of the restricted geographic sampling site and sampling period. However, measures were taken to address the underestimation of recent TB transmission by recruiting up to 90% of the diagnosed TB cases spanning a 3.5-year period. In addition, the possibility of overestimating recent TB transmission rates is also possible considering that the basis of the clustering analysis was done using combined 15-locus MIRU-VNTR typing and spoligotyping, whereas whole genome sequencing could have offered a better resolution of strains.

Overall, the findings indicate high recent TB transmission, suggesting the occurrence of unsuspected outbreaks. The intensification of community education is recommended to improve early case reporting and infection control.

## Funding

This research was funded by a Wellcome Trust Intermediate Fellowship Grant (097134/Z/11/Z) to Dorothy Yeboah-Manu. The funding source had no role in the study design, collection, analysis, and interpretation of the data, in the writing of the report, or in the decision to submit the paper for publication.

## Ethical approval

The Scientific and Technical Committee and then the Institutional Review Board at NMIMR, University of Ghana (FWA00001824) reviewed and approved the study.

## Conflict of interest

We declare that we have no competing interest.
