# Supplementary material for: Reduced transmission of Mycobacterium africanum compared to Mycobacterium tuberculosis in urban West Africa
Source: Int J Infect Dis. 2018 Aug;73:30–42. doi: 10.1016/j.ijid.2018.05.014 (PMC6069673; doi:10.1016/j.ijid.2018.05.014)
Supplement: Supplementary file 1 [file mmc1.docx]

**Reduced transmission of *Mycobacterium africanum* compared to *Mycobacterium tuberculosis* in urban West Africa**

Table S1: Summary of recruited cases

| **Parameters** | **Rural** | **Urban** | **Combined** |
| --- | --- | --- | --- |
| Number of pulmonary TB cases recruited | 382 (11·6%) | 2,921 (88·4%) | 3,303 |
| Number of MTBC isolates obtained | 254 (9·7%) | 2,350 (90·3%) | 2,604 (78·8%)* |
| Number of MTBC isolates passed for clustering analysis | 201 (8·7%) | 2,108 (91·3%) | 2,309 (88·7%)^#^ |
| Gender | 199 | 2,095 | 2,294 |
| Male | 151 (75·9%) | 1,480 (70·6%) | 1,631 (71·1%) |
| Female | 48 (24·1%) | 615 (29·4%) | 663 (28·9%) |
| Median age (range) | 37 (4 to 91) | 37 (3 to 90) | 37 (3 to 91) |
| Male | 38 (6 to 91) | 39 (3 to 89) | 39 (3 to 91) |
| Female | 33 (4 to 78) | 32 (4 to 90) | 33 (4 to 90) |

* Frequency expressed as a percentage of the total number of TB cases recruited

# Frequency expressed as the total number of MTBC isolates obtained

Table S2: Distribution of participants’ characteristics stratified by the main MTBC lineages found in Ghana: A univariate and multivariate logistic regression analysis

|  | **Distribution of Participants variables** | | | | **Maf lineage 5 vs MTBss L1-4** | | **Maf lineage 6 vs MTBss L1-4** | |
| --- | --- | --- | --- | --- | --- | --- | --- | --- |
| **Variables** | **All lineages (N=2,309)** | **MTBss L1 - 4 (N = 1,870)** | **Maf L5 (N = 289)** | **Maf L6 (N = 150)** | **Univariate** | **Multivariate** | **Univariate** | **Multivariate** |
|  | **N (%)** | **N (%)** | **N (%)** | **N (%)** | **OR (95% CI)** | **adj·OR (95% CI)** | **OR (95% CI)** | **adj·OR (95% CI)** |
| Gender | 2,294 (99·4) |  |  |  |  |  |  |  |
| Male | 1,631 (71·1) | 1,348 (72·6) | 183 (63·5) | 100 (67·6) | 0·7 (0·5 - 0·9)* | 0·7 (0·5 - 1·0)* | 0·8 (0·5 - 1·1) |  |
| Female | 663 (28·9) | 510 (27·5) | 105 (36·5) | 48 (32·4) | Reference |  | Reference |  |
| Age (yrs) | 2,224 (96·3) |  |  |  |  |  |  |  |
| <15 | 37 (1·7) | 30 (1·7) | 5 (1·8) | 2 (1·4) | 1·1 (0·4 (3·0 ) | 0·6 (0·2 - 2·3) | 1·0 (0·2 - 4·4) |  |
| 15 - 29 | 639 (28·7) | 526 (29·3) | 78 (27·6) | 35 (24·0) | Reference |  | Reference |  |
| 30 - 39 | 570 (25·6) | 465 (25·9) | 68 (24·0) | 37 (25·3) | 1·0 (0·7 - 1·4) | 0·8 (0·5 - 1·3) | 1·2 (0·7 - 1·9) |  |
| 40 - 59 | 778 (35·0) | 626 (34·9) | 94 (33·2) | 58 (39·7) | 1·0 (0·7 - 1·4) | 1·1 (0·7 - 1·7) | 1·4 (0·9 - 2·1) |  |
| >59 | 200 (9·0) | 148 (8·3) | 38 (13·4) | 14 (9·6) | 1·7 (1·1 - 2·7)* | 1·5 (0·7 - 2·9) | 1·4 (0·7 - 2·7) |  |
| Nationality | 1,781 (77·1) |  |  |  |  |  |  |  |
| Ghanaian | 1,714 (96·2) | 1,378 (96·0) | 227 (97·4) | 109 (96·5) | Reference |  | Reference |  |
| Others | 67 (3·8) | 57 (4·0) | 6 (2·6) | 4 (3·5) | 0·6 (0·3 - 1·5) |  | 0·9 (0·3 - 2·5) |  |
| Locality | 2,309 (100) |  |  |  |  |  |  |  |
| Rural | 201 (8·7) | 172 (9·2) | 15 (5·2) | 14 (9·3) | Reference |  | Reference |  |
| Urban | 2,108 (91·3) | 1,698 (90·8) | 274 (94·8) | 136 (90·7) | 1·9 (1·1 - 3·2)* |  | 1·0 (0·6 - 1·7) |  |
| Residence classification | 1,642 (71·1) |  |  |  |  |  |  |  |
| Village | 69 (4·2) | 56 (4·2) | 4 (1·9) | 9 (8·3) | 0·4 (0·2 - 1·3) |  | 2·1 (1·0 - 4·4)* | 6·6 (1·2 - 36·1)* |
| Town | 182 (11·1) | 145 (10·9) | 24 (11·6) | 13 (11·9) | 1·0 (0·6 - 1·7) |  | 1·2 (0·6 - 2·2) | 1·6 (0·7 - 3·9) |
| City residential area | 52 (3·2) | 39 (2·9) | 10 (4·8) | 3 (2·8) | 1·6 (0·8 - 3·3) |  | 1·0 (0·3 - 3·3) | 1·1 (0·2 - 5·2) |
| City suburb | 1,136 (69·2) | 920 (69·4) | 146 (70·5) | 70 (64·2) | Reference |  | Reference |  |
| City Slum | 203 (12·4) | 166 (12·5) | 23 (11·1) | 14 (12·8) | 0·9 (0·5 - 1·4) |  | 1·1 (0·6 - 2·0) | 0·9 (0·4 - 1·7) |
| Residential district | 1,538 (66·6) |  |  |  |  |  |  |  |
| Ablekuma | 545 (35·4) | 438 (35·1) | 77 (40·7) | 30 (29·1) |  |  | Reference |  |
| Ashiedu Keteke | 170 (11·1) | 136 (10·9) | 13 (6·9) | 21 (20·4) | 0·5 (0·3 - 1·0) |  | 2·3 (1·2 - 4·0)* | 2·2 (1·1 - 4·4)* |
| Ayawaso | 220 (14·3) | 185 (14·8) | 21 (11·1) | 14 (13·6) | 0·6 (0·4 - 1·1) |  | 1·1 (0·6 - 2·1) | 0·9 (0·4 - 1·9) |
| Kpeshie | 224(14·6) | 179 (14·4) | 37 (19·6) | 8 (7·8) | 1·2 (0·8 - 1·8) |  | 0·6 (0·3 - 1·4) | 0·4 (0·1 - 1·2) |
| Mamprusi East | 70 (4·6) | 60 (4·8) | 4 (2·1) | 6 (5·8) | 0·4 (0·1 - 1·0) |  | 1·4 (0·6 - 3·6) | 0·24 (0·04 - 1·55) |
| Okaikoi | 176 (11·4) | 139 (11·2) | 24 (12·7) | 13 (12·6) | 1·0 (0·6 - 1·6) |  | 1·6 (0·7 - 2·7) | 1·3 (0·6 - 2·8) |
| Osu Klottey | 133 (8·6) | 109 (8·8) | 13 (69) | 11 (10·7) | 0·7 (0·4 - 1·3) |  | 1·5 (0·7 - 3·0) | 1·6 (0·7 - 3·5) |
| Education | 1,748 (75·7) |  |  |  |  |  |  |  |
| Primary | 222 (12·7) | 180 (12·8) | 23 (10·1) | 19 (17·0) | 0·7 (0·4 - 1·1) |  | 1·8 (1·0 - 3·3)* | 1·5 (0·7 - 3·1) |
| Middle/JHS | 637 (36·4) | 514 (36·5) | 93 (41·0) | 30 (26·8) | Reference |  | Reference |  |
| Secondary | 429 (24·5) | 348 (24·7) | 55 (24·2) | 26 (23·21) | 0·9 (0·6 - 1·3) |  | 1·3 (0·7 - 2·2) | 1·2 (0·6 - 2·2) |
| Tertiary | 190 (10·9) | 151 (10·7) | 27 (11·9) | 12 (10·7) | 1·0 (0·6 - 1·6) |  | 1·4 (0·7 - 2·7) | 1·9 (0·8 - 4·4) |
| No Education | 270 (15·4) | 216 (15·3) | 29 (12·8) | 25 (22·3) | 0·7 (0·5 -1·2) |  | 2·0 (1·1 - 3·4)* | 1·8 (0·9 - 3·7) |
| Occupation | 1,722 (74·6) |  |  |  |  |  |  |  |
| Unemployed | 390 (22·6) | 311 (22·4) | 60 (27·0) | 19 (16·8) | 1·2 (0·9 - 1·7) |  | 0·7 (0·4 - 1·2) |  |
| Unskilled | 951 (55·2) | 765 (55·2) | 118 (53·1) | 68 (60·2) | Reference |  | Reference |  |
| Skilled | 381 (22·1) | 311 (22·4) | 44 (19·8) | 23 (23·0) | 0·9 (0·6 - 1·3) |  | 0·9 (0·6 - 1·5) |  |
| Religion | 1,771 (76·7) |  |  |  |  |  |  |  |
| Christian | 1,361 (76·9) | 1,081 (75·8) | 198 (84·6) | 82 (73·9) | Reference |  | Reference |  |
| Islam | 302 (17·0) | 254 (17·8) | 29 (12·4) | 19 (17·1) | 0·6 (0·4 - 0·9)* |  | 1·0 (0·6 - 1·6) |  |
| Others | 26 (1·5) | 20 (1·4) | 3 (1·3) | 3 (2·7) | 0·8 (0·2 - 2·8) |  | 2·0 (0·6 - 6·8) |  |
| Not religious | 82 (4·6) | 71 (50) | 4 (1·7) | 7 (6·3) | 0·3 (0·1 - 0·8)* |  | 1·3 (0·6 - 2·9) |  |
| Ethnicity | 1,760 (76·4) |  |  |  |  |  |  |  |
| Akan | 570 (32·3) | 458 (32·2) | 90 (39·3) | 22 (19·6) | Reference |  | Reference |  |
| Ewe | 259 (14·7) | 204 (14·3) | 35 (15·3) | 20 (17·9) | 0·9 (0·6 - 1·3) |  | 2·0 (1·1 - 3·8)* | 2·3 (1·1 - 5·1)* |
| Ga/Adangbe | 544 (30·8) | 437 (30·7) | 71 (31·0) | 36 (32·1) | 0·8 (0·6 - 1·2) |  | 1·7 (1·0 - 3·0) | 1·8 (0·9 - 3·6) |
| Others | 392 (22·2) | 325 (22·8) | 33 (14·4) | 34 (30·4) | 0·5 (0·3 - 0·8) |  | 2·2 (1·2 - 3·8)* | 1·6 (0·7 - 3·4) |
| Marital Status | 1,758 (76·1) |  |  |  |  |  |  |  |
| Single | 766 (43·6) | 618 (43·6) | 101 (44·3) | 47 (41·2) | Reference |  | Reference |  |
| Married | 742 (42·2) | 599 (42·3) | 93 (40·8) | 50 (43·9) | 0·9 (0·7 - 1·3) |  | 1·1 (0·7 - 1·7) |  |
| Divorced | 167 (9·5) | 137 (9·7) | 19 (8·3) | 11 (9·6) | 0·8 (0·5 - 1·4) |  | 1·1 (0·5 - 2·1) |  |
| Widowed | 83 (4·7) | 62 (4·4) | 15 (6·6) | 6 (5·3) | 1·5 (0·8 - 2·7) |  | 1·3 (0·5 - 3·1) |  |
| Smear positivity | 2,208 (95·6) |  |  |  |  |  |  |  |
| Scanty 1 - 9 | 173 (7·8) | 147 (8·2) | 18 (6·6) | 8 (5·6) | 0·8 (0·5 - 1·4) |  | 0·6 (0·3 - 1·3) |  |
| 1+ | 474 (21·5) | 381 (21·2) | 67 (24·6) | 26 (18·3) | 1·5 (0·9 - 1·6) |  | 0·8 (0·5 - 1·2) |  |
| 2+ | 546 (24·7) | 445 (24·8) | 65 (23·9) | 36 (25·4) | 1·0 (0·7 - 1·4) |  | 0·9 (0·6 - 1·4) |  |
| 3+ | 1,015 (46·0) | 821 (45·8) | 122 (44·9) | 72 (50·7) | Reference |  | Reference |  |
| Previous TB treatment | 1,737 (75·2) |  |  |  |  |  |  |  |
| Yes | 291 (16·8) | 242 (17·2) | 30 (13·5) | 19 (17·0) | 0·7 (0·5 - 1·1) |  | 1·0 (0·6 - 1·6) |  |
| No | 1,446 (83·2) | 1,161 (82·8) | 192 (86·5) | 93 (83·0) | Reference |  | Reference |  |
| Outcome of TB Treatment | 151 (51·9) |  |  |  |  |  |  |  |
| Cured | 79 (52·3) | 69 (54·3) | 8 (44·4) | 2 (33·3) | Reference |  | Reference |  |
| Not cured | 36 (23·8) | 28 (22·1) | 6 (33·3) | 2 (33·3) | 1·8 (0·6 - 5·8) |  | 2·5 (0·3 - 18·4) |  |
| Defaulted | 36 (23·8) | 30 (23·6) | 4 (22·2) | 2 (33·3) | 1·1 (0·3 - 4·1) |  | 2·3 (0·3 - 17·0) |  |
| Risk of TB contact |  |  |  |  |  |  |  |  |
| Closefriend/Household | 1,665 (72·1) |  |  |  |  |  |  |  |
| Any | 544 (32·7) | 442 (33·0) | 62 (29·1) | 40 (35·7) | 0·8 (0·6 - 1·1) |  | 1·1 (0·7 - 1·7) |  |
| No | 1,121 (67·3) | 898 (67·0) | 151 (70·9) | 72 (64·3) | Reference |  | Reference |  |
| Closefriend/Household | 1,665 (72·1) |  |  |  |  |  |  |  |
| No contact | 1,121 (67·3) | 898 (67·0) | 151 (70·9) | 72 (64·3) | Reference |  | Reference |  |
| 1 contact | 212 (12·7) | 179 (13·4) | 20 (9·4) | 13 (11·6) | 0·7 (0·4 - 1·1) |  | 0·9 (0·5 - 1·7) |  |
| 2-5 contacts | 309 (18·6) | 243 (18·1) | 39 (18·3) | 27 (24·1) | 0·9 (0·6 - 1·4) |  | 1·4 (0·9 - 2·2) |  |
| 6-10 contacts | 23 (1·4) | 20 (1·5) | 3 (1·4) | 0 | 0·9 (0·3 - 3·0) |  |  |  |
| Imprisonment | 1,660 (71·9) |  |  |  |  |  |  |  |
| Yes | 97 (5·8) | 81 (6·1) | 15 (7·1) | 1 (0·9) | 1·2 (0·7 - 2·1) |  |  |  |
| No | 1,563 (94·2) | 1,257 (93·9) | 196 (92·9) | 110 (99·1) | Reference |  |  |  |
| Health/Lab worker | 1,661 (71·9) |  |  |  |  |  |  |  |
| Yes | 47 (2·8) | 40 (3·0) | 5 (2·4) | 2 (1·8) | 0·8 (0·3 - 2·0) |  | 0·6 (0·1 - 2·5) |  |
| No | 1,614 (97·2) | 1,299 (97·0) | 206 (97·6) | 109 (98·2) | Reference |  | Reference |  |
| Immunosuppressive condition | 1,695 (73·4) |  |  |  |  |  |  |  |
| Any | 893 (52·7) | 720 (52·9) | 111 (49·8) | 62 (56·4) | 0·9 (0·7 - 1·2) |  | 1·1 (0·8 - 1·7) |  |
| None | 802 (47·3) | 642 (47·1) | 112 (50·2) | 48 (43·6) | Reference |  | Reference |  |
| Diabetes Mellitus | 534 (23·1) |  |  |  |  |  |  |  |
| Yes | 104 (19·5) | 78 (18·1) | 18 (24·7) | 8 (25·8) | 1·5 (0·8 - 2·6) |  | 1·6 (0·7 - 3·6) |  |
| No | 430 (80·5) | 352 (81·9) | 55 (75·3) | 23 (74·2) | Reference |  | Reference |  |
| HIV status | 1,166 (50·5) |  |  |  |  |  |  |  |
| Yes | 144 (12·3) | 117 (12·4) | 20 (13·7) | 7 (9·2) | 1·1 (0·7 - 1·9) |  | 0·7 (0·3 - 1·6) |  |
| No | 1,022 (87·7) | 827 (87·6) | 126 (86·3) | 69 (90·8) | Reference |  | Reference |  |
| Smoking | 1,518 (65·7) |  |  |  |  |  |  |  |
| Yes | 434 (28·6) | 366 (30·1) | 37 (18·6) | 31 (29·8) | 0·5 (0·4 - 0·8)* |  | 1·0 (0·6 - 1·5) |  |
| No | 1,084 (71·4) | 849 (69·9) | 162 (81·4) | 73 (70·2) | Reference |  | Reference |  |
| Substance abuse (including acohol) | 1,474 (63·8) |  |  |  |  |  |  |  |
| Yes | 460 (31·2) | 375 (31·7) | 57 (29·5) | 28 (28·3) | 0·9 (0·6 - 1·3) |  | 0·8 (0·5 - 1·3) |  |
| No | 1,014 (68·8) | 807 (68·3) | 136 (70·5) | 71 (71·7) | Reference |  | Reference |  |
| Drug resistance | 2,300 (99·6) |  |  |  |  |  |  |  |
| Any | 313 (13·6) | 258 (13·8) | 37 (12·8) | 18 (12·2) | 0·9 (0·6 - 1·3) |  | 0·9 (0·5 - 1·4) |  |
| None | 1,987 (86·4) | 1,606 (86·2) | 251 (87·2) | 130 (87·8) | Reference |  | Reference |  |
| Cluster size (number of isolates) | 1,227 (53·1) |  |  |  |  |  |  |  |
| Small (2) | 290 (23·6) | 208 (20·1) | 74 (51·7) | 8 (16·7) | Reference |  | Reference |  |
| Medium (3 - 5) | 262 (21·4) | 216 (20·9) | 35 (24·5) | 11 (22·9) | 0·5 (0·3 - 0·7)* | 0·5 (0·3 - 0·7)* | 1·3 (0·5 - 3·4) |  |
| Large (6 - 20) | 452 (36·8) | 389 (37·5) | 34 (23·8) | 29 (60·4) | 0·2 (0·2 - 0·3)** | 0·2 (0·1 - 0·4)** | 1·9 (0·9 - 4·3) |  |
| Very large (>20) | 223 (18·2) | 223 (21·5) | 0 | 0 |  |  |  |  |

For the multivariate model, we included only variables with p<0·1

*p<0·05 **p<0·001


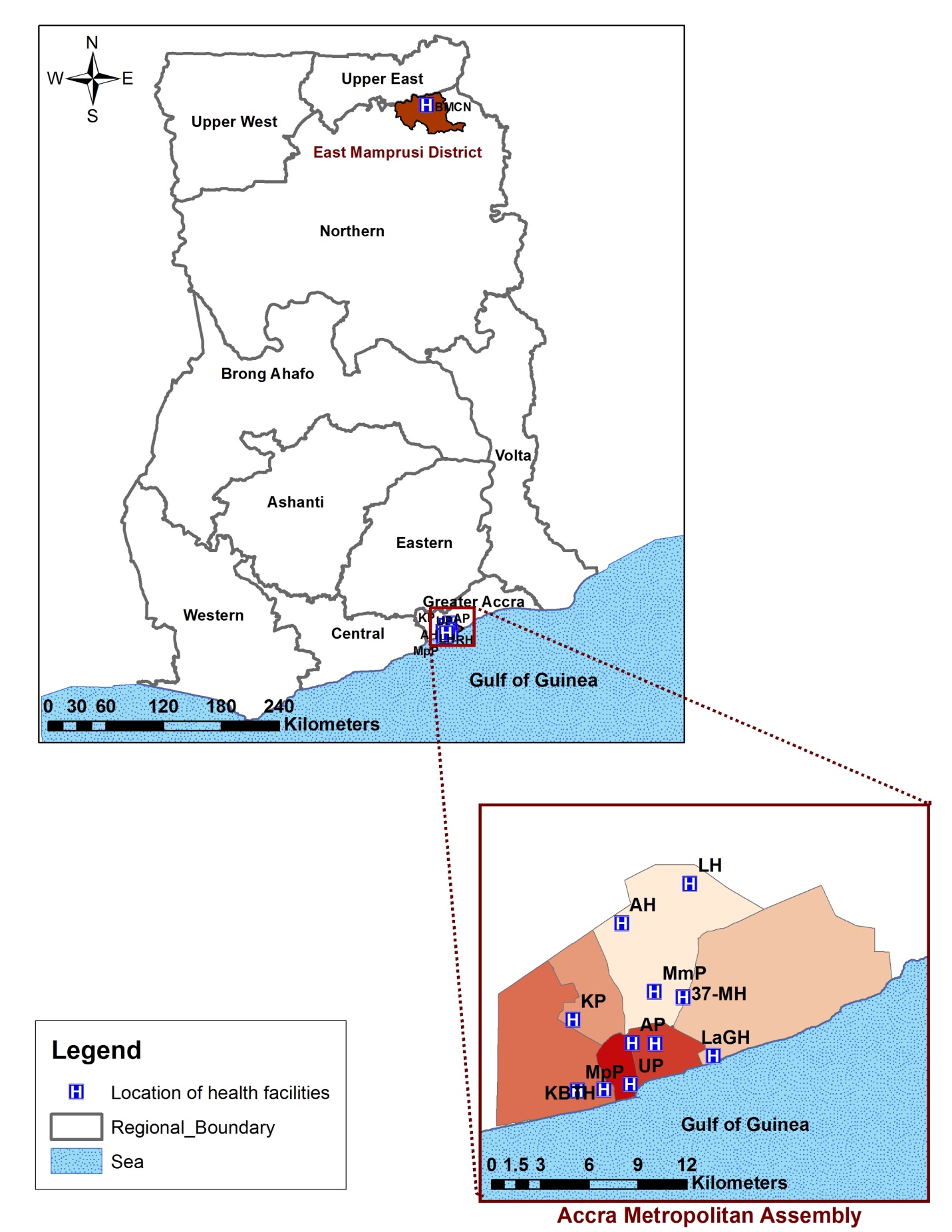


**Figure S1: Map of study area showing the location of the 12 sampling sites (health facilities)**


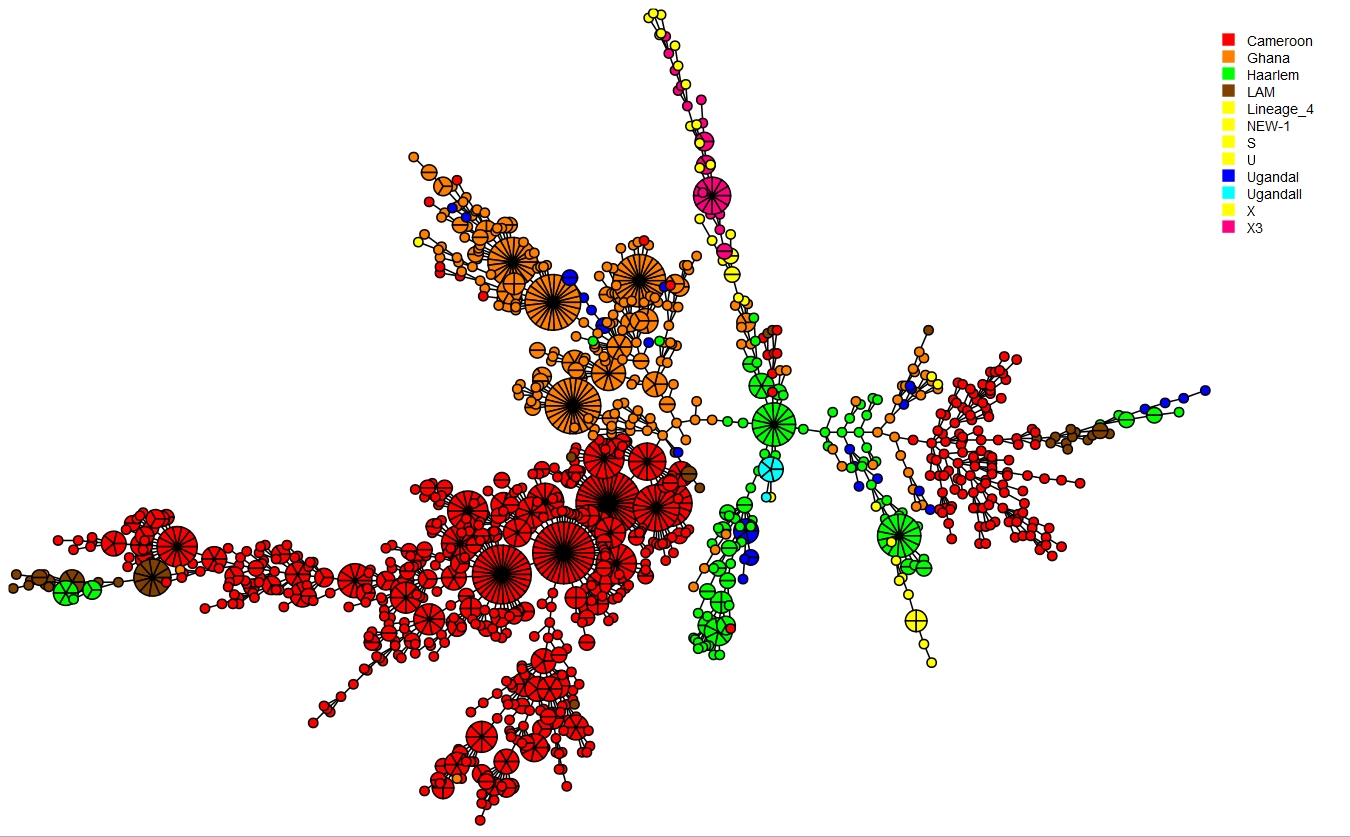


Figure S2: Minimum spanning tree (MST) representation of the clustering of sub-lineages of lineage 4 MTBC isolates built with Bionumerics software. The size of the clusters shows the number of clustered isolates within the cluster with the same strain type. The color codes indicate the various L4 sub-lineages and have been provided in the figure legend.


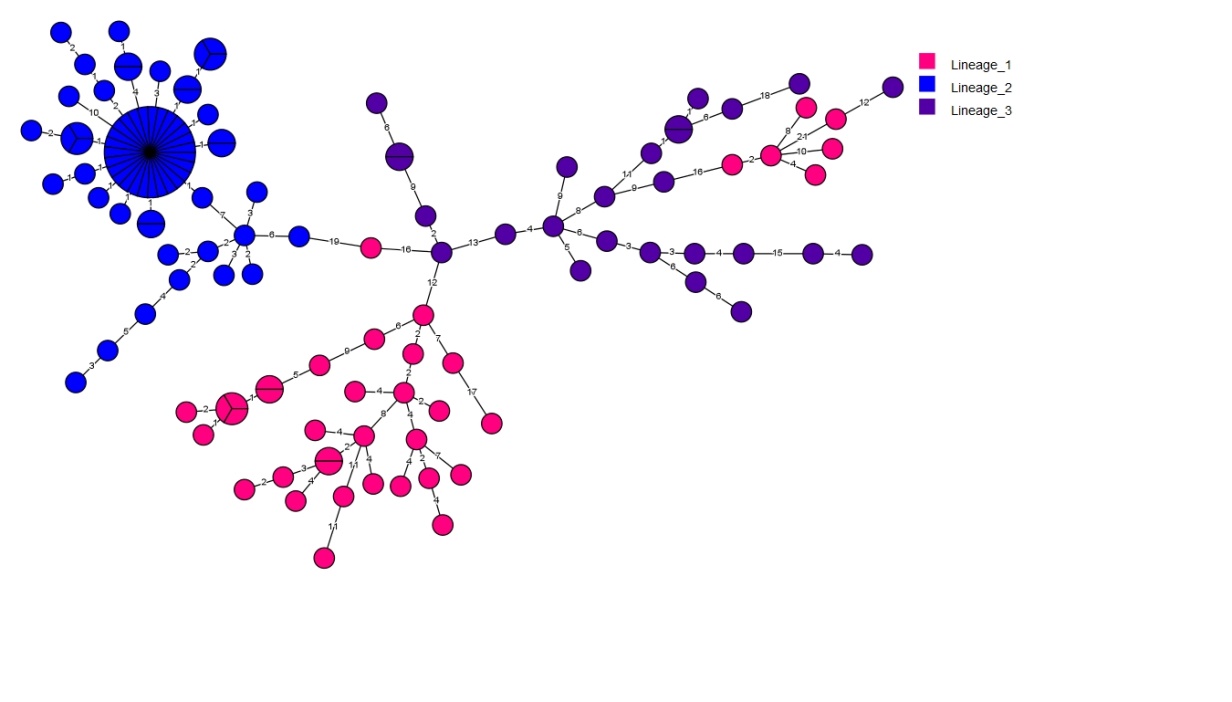


Figure S3: MST representation of the clustering of lineage 1 - 3 MTBC isolates built with Bionumerics software**.** The size of the clusters shows the number of clustered isolates within the cluster with the same strain type. Digits on the branches indicate number of polymorphic VNTR/Spoligo-spacer between isolates among the linked cases.


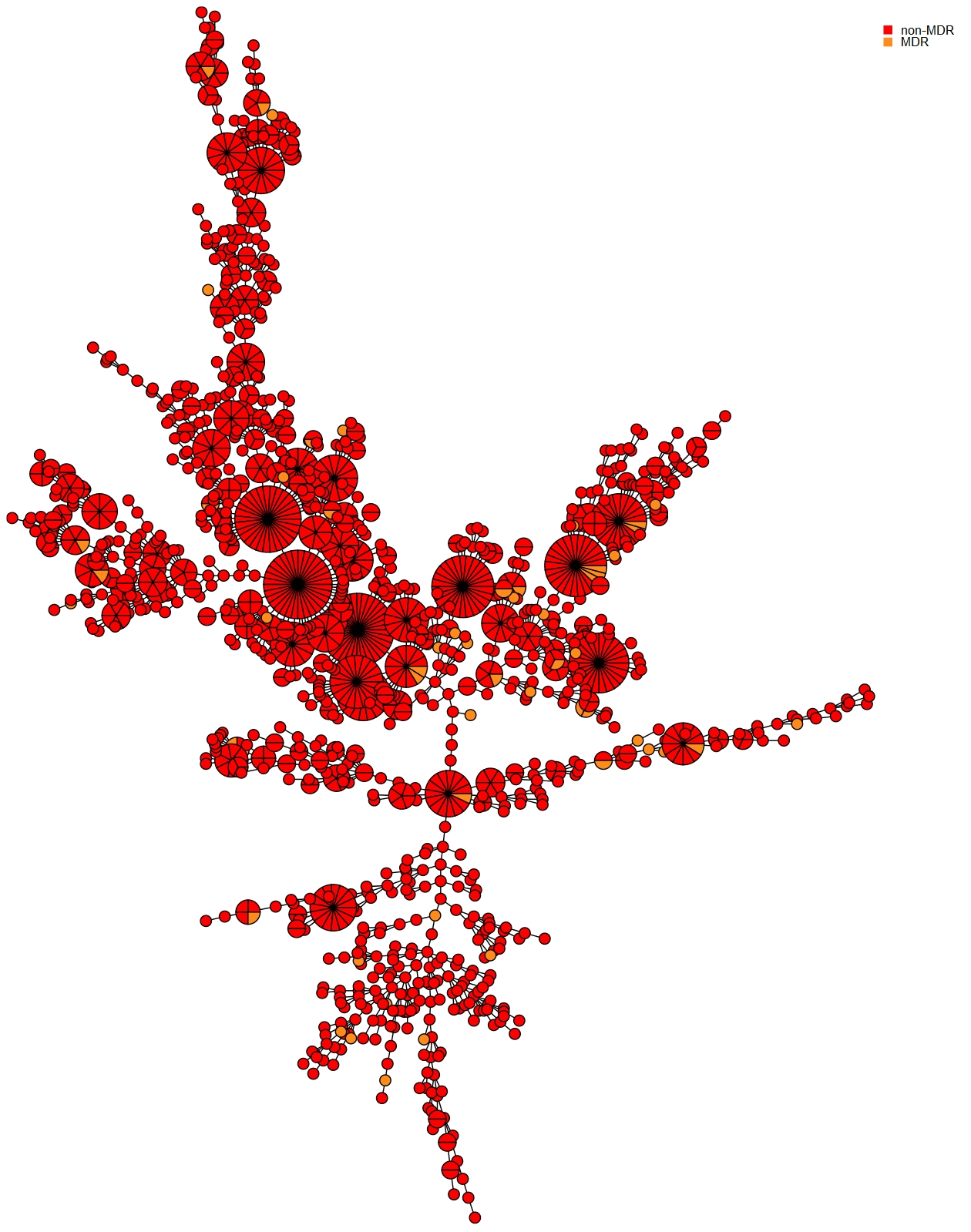


Figure S4: MST representation of the clustering of lineage 4 MTBC isolates built with Bionumerics software**.** The size of the clusters shows the number of clustered isolates within the cluster with the same strain type. Orange circles or sectors indicate individual cases with multidrug resistant (MDR) TB strains.


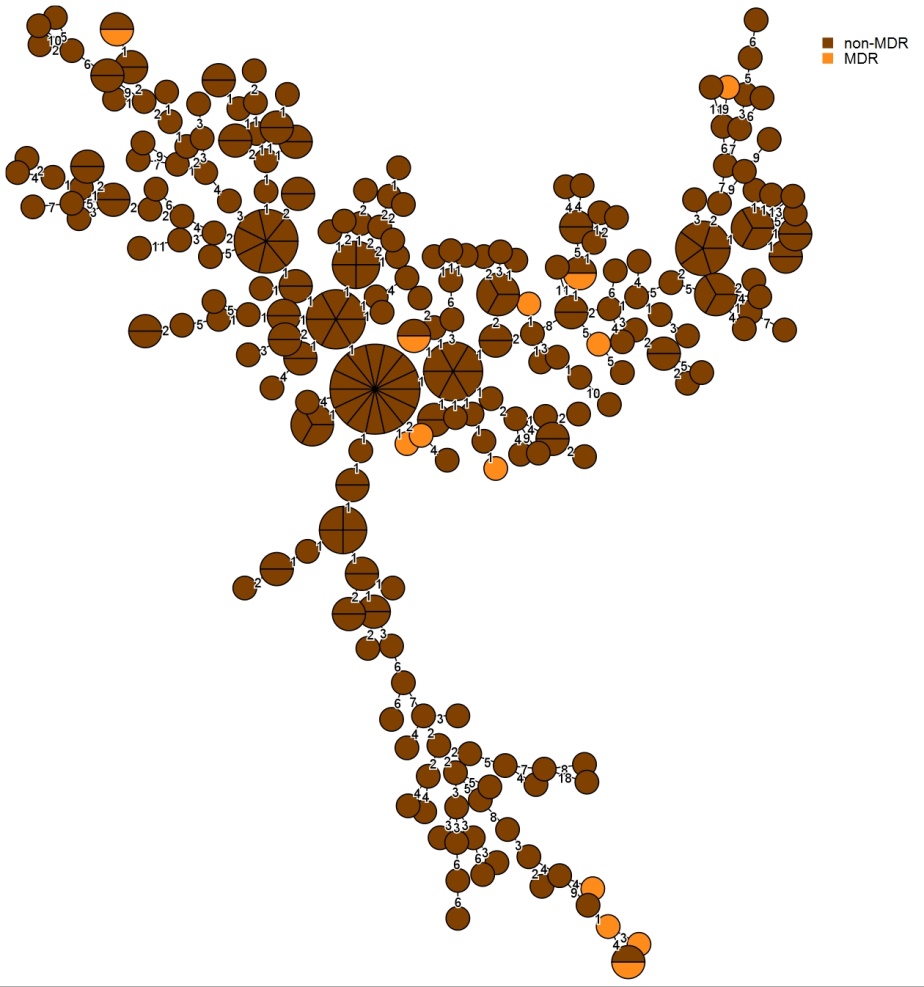


Figure S5: MST representation of the clustering of lineage 5 MTBC isolates built with Bionumerics software**.** The size of the clusters shows the number of clustered isolates within the cluster with the same strain type. Digits on the branches indicate number of polymorphic VNTR/Spoligo-spacer between isolates among the linked cases. Orange circles or sectors indicate individual cases with multidrug resistant (MDR) TB strains.


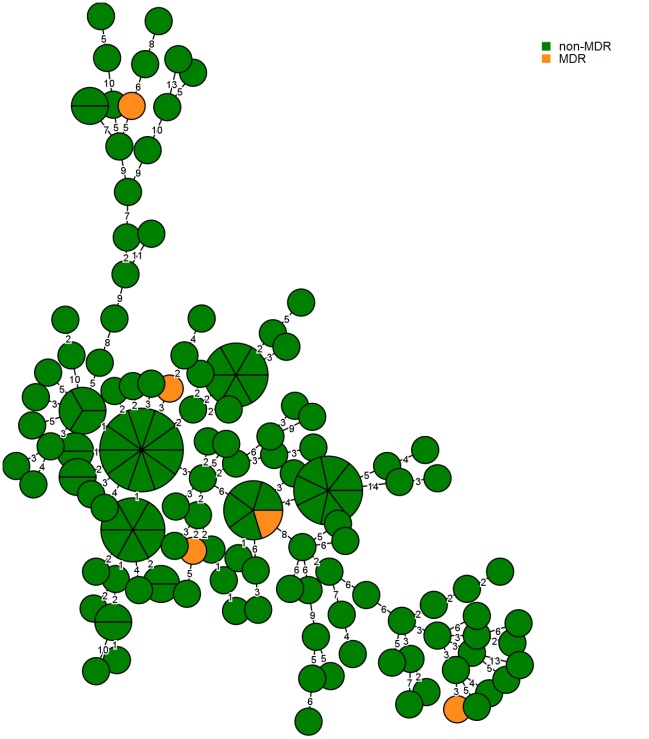


Figure S6: MST representation of the clustering of lineage 6 MTBC isolates built with Bionumerics software**.** The size of the clusters shows the number of clustered isolates within the cluster with the same strain type. Digits on the branches indicate number of polymorphic VNTR/Spoligo-spacer between isolates among the linked cases. Orange circles or sectors indicate individual cases with multidrug resistant (MDR) TB strains.


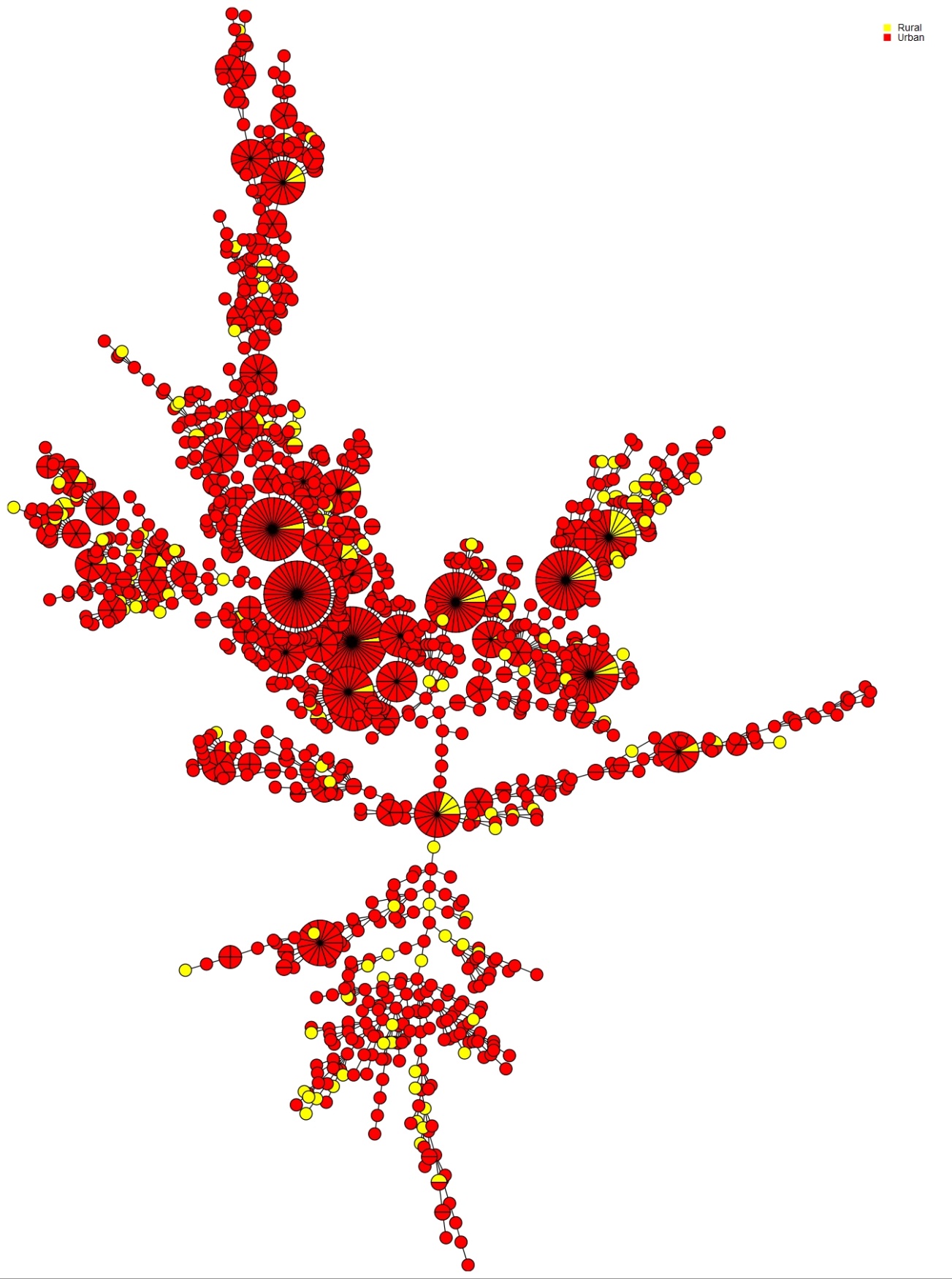


Figure S7: MST representation of the clustering of lineage 4 MTBC isolates stratified by study setting. The size of the clusters shows the number of clustered isolates within the cluster with the same strain type. Yellow circles or sectors indicate individual cases form the rural setting.


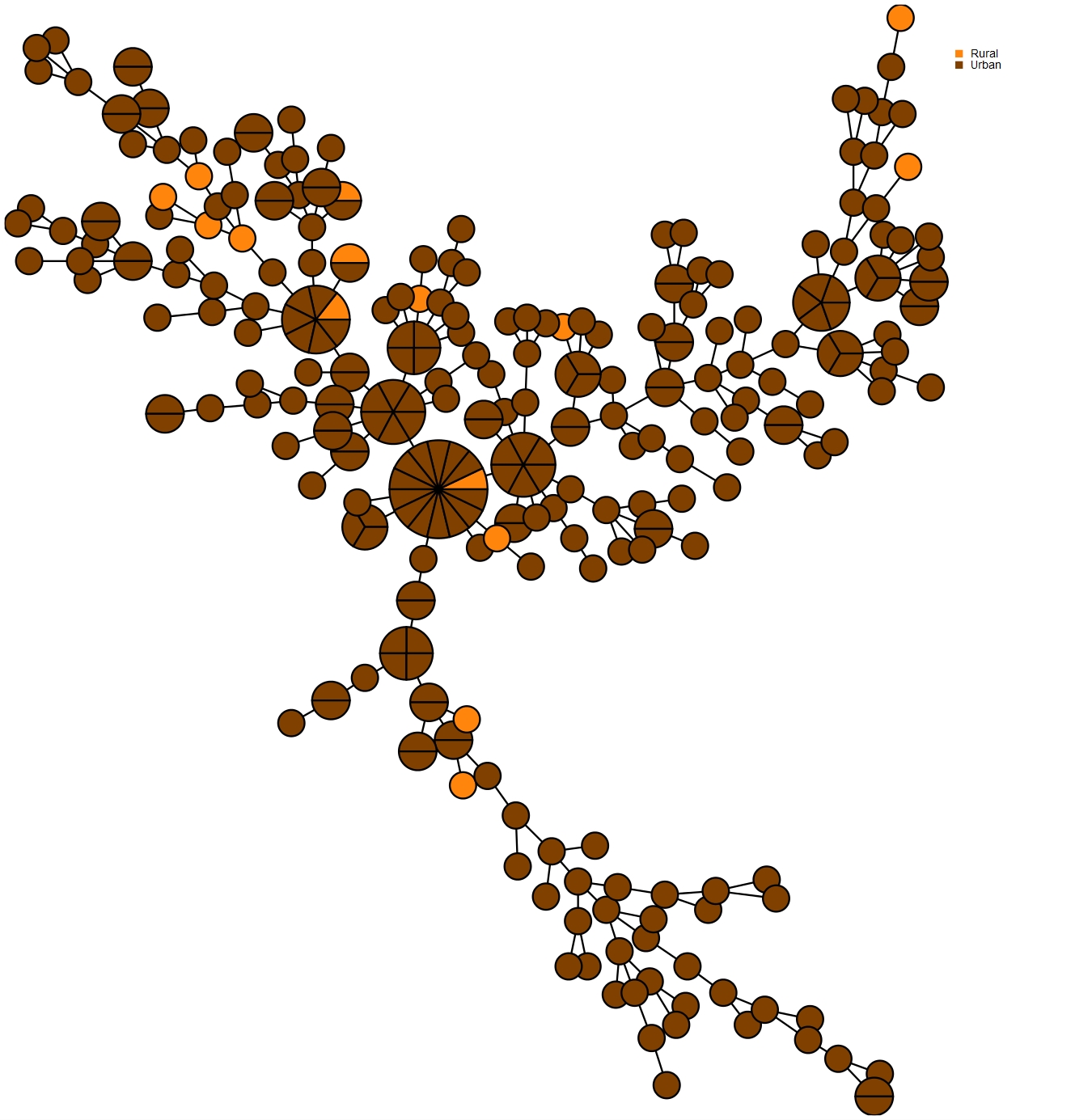


Figure S8: MST representation of the clustering of lineage 5 MTBC isolates stratified by study setting. The size of the clusters shows the number of clustered isolates within the cluster with the same strain type. Orange circles or sectors indicate individual cases form the rural setting.


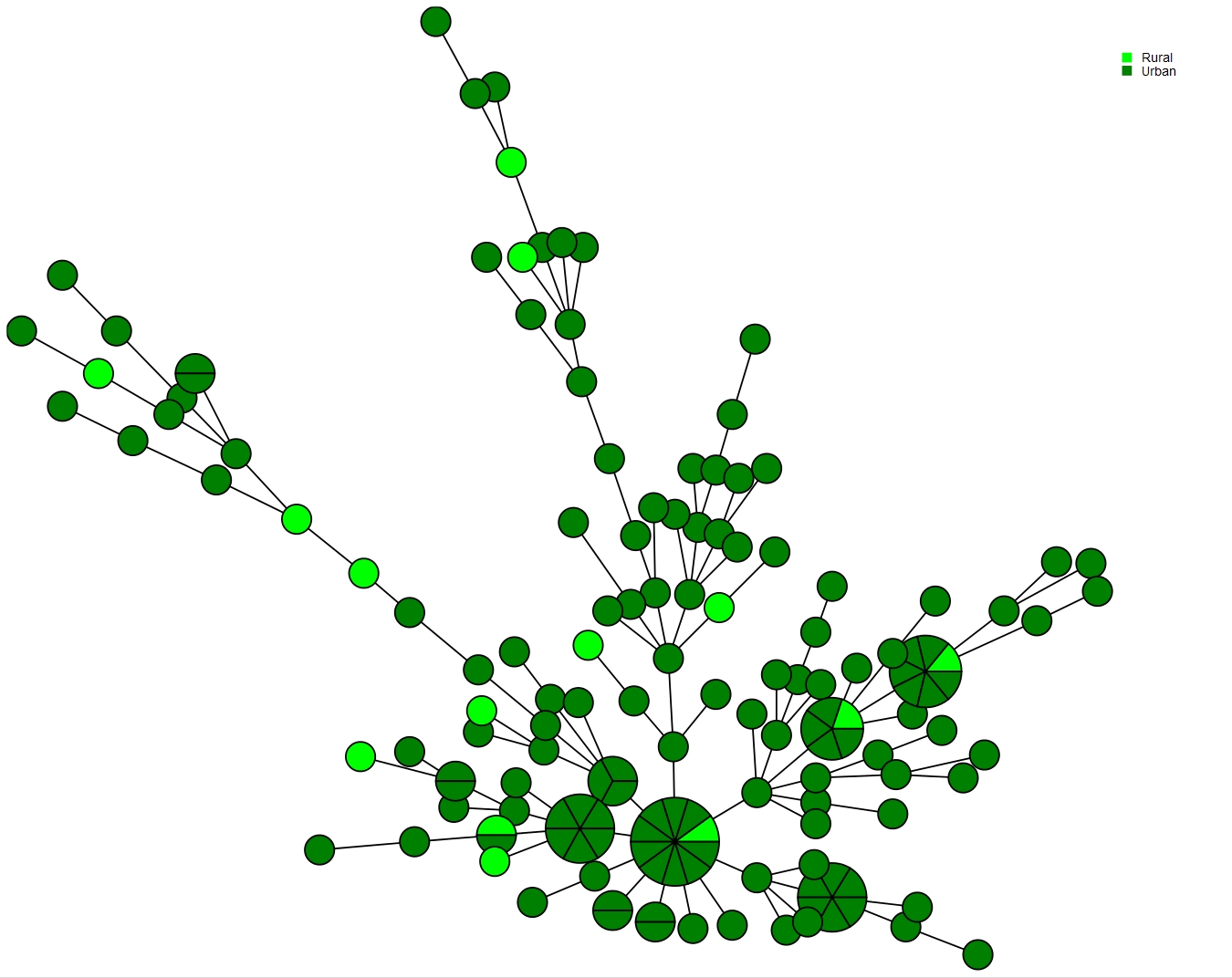


Figure S9: MST representation of the clustering of lineage 6 MTBC isolates stratified by study setting. The size of the clusters shows the number of clustered isolates within the cluster with the same strain type. Light green circles or sectors indicate individual cases form the rural setting.


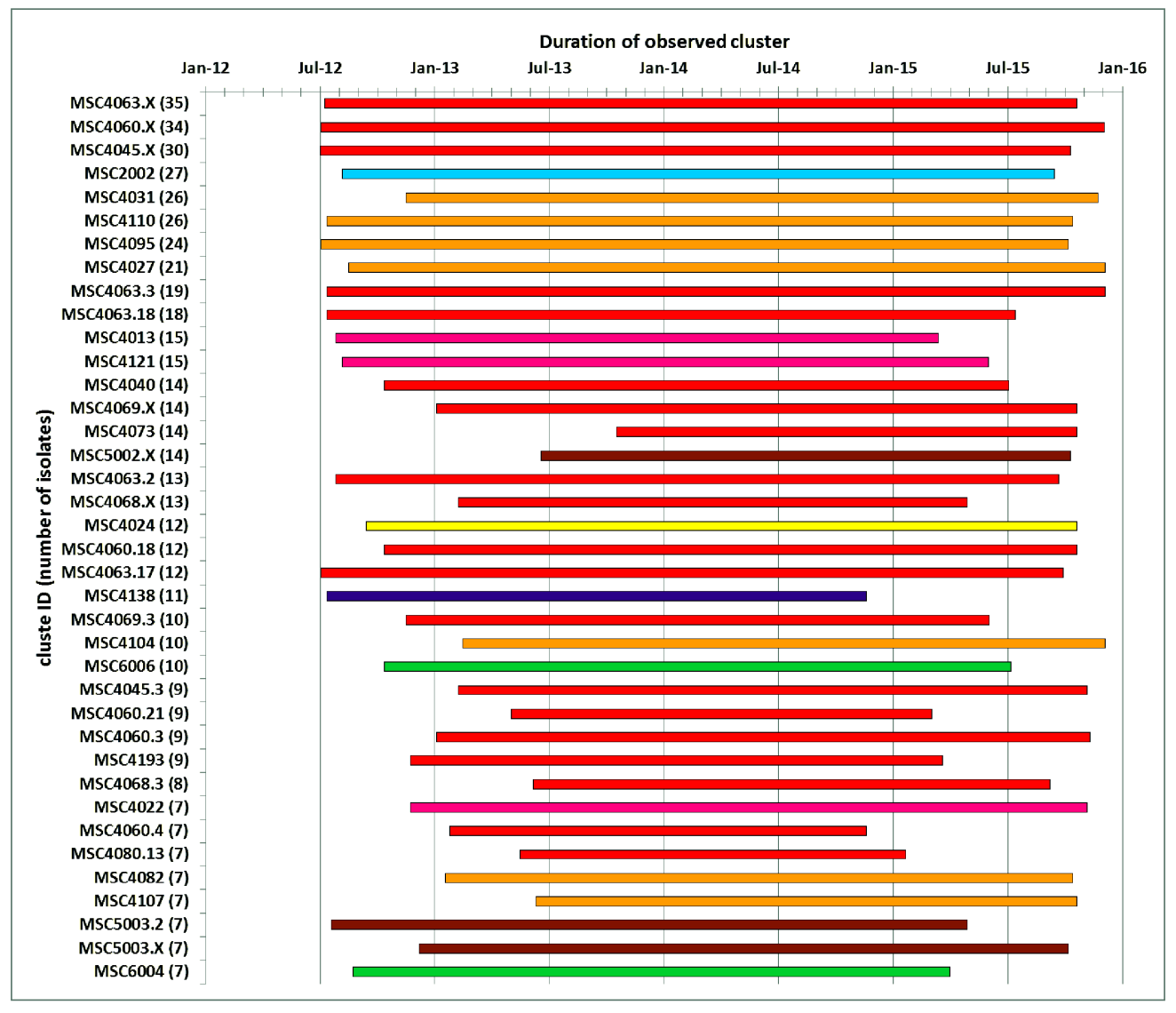


**Figure S10: Time lapse between first and last diagnosed case within selected large and very large clusters**. Lineages 5 and 6 have been color coded with the universally accepted color codes for the main MTBC lineages whereas sub-lineages of lineage 4 have been color coded, Cameroon: red, Ghana: gold, Haarlem: pink, LAM: purple and X3: yellow.
